# Polysaccharide monooxygenase-catalyzed oxidation of cellulose to glucuronic acid-containing cello-oligosaccharides

**DOI:** 10.1186/s13068-019-1384-0

**Published:** 2019-02-27

**Authors:** Jinyin Chen, Xiuna Guo, Min Zhu, Chen Chen, Duochuan Li

**Affiliations:** Department of Mycology, Shandong Agricultural University, Taian, 271018 Shandong China

**Keywords:** *Humicola insolens*, Polysaccharide monooxygenase (PMO), Glucuronic acid-containing cello-oligosaccharides, C6 oxidation, Glucuronic acid, Saccharic acid

## Abstract

**Background:**

Polysaccharide monooxygenases (PMOs) play an important role in the enzymatic degradation of cellulose. They have been demonstrated to able to C6-oxidize cellulose to produce C6-hexodialdoses. However, the biological function of C6 oxidation of PMOs remains unknown. In particular, it is unclear whether C6-hexodialdoses can be further oxidized to uronic acid (glucuronic acid-containing oligosaccharides).

**Results:**

A PMO gene, *Hipmo1*, was isolated from *Humicola insolens* and expressed in *Pichia pastoris*. This PMO (HiPMO1), belonging to the auxiliary activity 9 (AA9) family, was shown to able to cleave cellulose to yield non-oxidized and oxidized cello-oligosaccharides. The enzyme oxidizes C6 positions in cellulose to form glucuronic acid-containing cello-oligosaccharides, followed by hydrolysis with beta-glucosidase and beta-glucuronidase to yield glucose, glucuronic acid, and saccharic acid. This indicates that HiPMO1 can catalyze C6 oxidation of hydroxyl groups of cellulose to carboxylic groups.

**Conclusions:**

HiPMO1 oxidizes C6 of cellulose to form glucuronic acid-containing cello-oligosaccharides followed by hydrolysis with beta-glucosidase and beta-glucuronidase to yield glucose, glucuronic acid, and saccharic acid, and even possibly by beta-eliminative cleavage to produce unsaturated cello-oligosaccharides. This study provides a new mechanism for cellulose cleavage by C6 oxidation of HiPMO1.

**Electronic supplementary material:**

The online version of this article (10.1186/s13068-019-1384-0) contains supplementary material, which is available to authorized users.

## Background

Cellulose, as the most abundant renewable biomass on earth, is a promising feedstock for fuel production. Considering the wide use of ethanol as an alternative fuel around the world, converting cellulose into ethanol through enzymatic breakdown of cellulose followed by fermentation has great potential for ethanol fuel production [1–4]. Currently, both hydrolytic and oxidative mechanisms have been proposed for the enzymatic degradation of cellulose [5]. The hydrolytic degradation of cellulose requires the cooperative action of 3 classes of cellulases: endoglucanases, exoglucanases/cellobiohydrolases, and beta-glucosidases [6, 7], while the oxidative degradation of cellulose is catalyzed by Cu^2+^-dependent polysaccharide monooxygenases (PMOs) that cleave glycosidic bonds of cellulose by an oxidative mechanism [8–15].

Based on the recent Carbohydrate-Active Enzymes (CAZy) database (www.cazy.org), 15 families exist in the auxiliary activity (AA) group. Five AA families (AA9, AA10, AA11, AA13, AA14, and AA15) comprise PMOs. Among the PMOs, AA9 PMOs are currently receiving significant attention due to their roles in the oxidative degradation of crystalline cellulose [5, 10, 13–24]. From data of the CAZy database, sixteen structures of AA9 PMOs are available (www.cazy.org). The AA9 PMO crystal structures reveal a relatively flat substrate-binding surface that is thought to interact with crystalline cellulose. The copper ion lies in the active center of the flat substrate-binding surface. There are two highly conserved histidine residues that directly coordinate with Cu^2+^ to form a structure called histidine brace [8, 10, 11, 13, 25]. The AA9 PMO enzymes can oxidize the C1, C4, and C6 carbon atoms of the glucose unit of cellulose. The oxidation at C1 and C4 leads to direct cleavage of the glucosidic bond of cellulose to yield aldonic acids and C4-ketoaldoses, respectively [5, 13, 17, 23, 26–28]. The C6 oxidation results in the formation of C6-hexodialdoses [10, 21, 29].

Although PMOs have been demonstrated to able to C6 oxidize cellulose to produce C6-hexodialdoses [10, 21, 29], the biological function of PMO C6 oxidation remains unclear because it cannot cause direct cleavage of the glucosidic cellulose bond, differing from C1 and C4 oxidation. In particular, it is unclear whether C6-hexodialdoses can be further oxidized to uronic acid (glucuronic acid-containing oligosaccharides). It was previously hypothesized that an unknown mechanism might oxidize C6-hexodialdoses to glucuronic acid-containing cello-oligosaccharides [29]. However, currently no evidence supports this hypothesis.

Thermophilic fungi are a class of extremophilic eukaryotic organisms growing in high temperatures of up to 60 °C. They are a potential reservoir of thermostable proteins for biochemical and structural analyses and industrial applications [30–33]. Here, we provide evidence that a C6-oxidizing AA9 PMO (HiPMO1) from the thermophilic fungus *Humicola insolens* (*Scytalidium thermophilum*) can C6-oxidize cellulose to form glucuronic acid-containing cello-oligosaccharides.

## Methods

### Plasmids, strains, enzymes, chemicals, and culture media

We isolated the *Humicola insolens* (*Scytalidium thermophilum*) CGMCC3.18482 strain (hereafter *H. insolens*) from horse dung in China and deposited the strain in the publicly accessible culture collection, the China General Microbiological Culture Collection Center (CGMCC). For total RNA isolation, *H*. *insolens* was cultured as previously described [29]. The *Pichia pastoris* GS115 strain (hereafter *P. pastoris*) and the plasmid vector pPICZαA were purchased from Invitrogen. Cellodextrin oligosaccharide mixture was purchased from Elicityl (Crolles, France). Avicel PH-101, ascorbate (Vc), glucose, gluconic acid, gluconic acid lactone, glucuronic acid, glucuronolactone, saccharic acid, beta-glucuronidase from bovine liver, and beta-glucosidase from almonds were purchased from Sigma-Aldrich.

### cDNA cloning, expression vector construction, and *P*. *pastoris* transformation

We isolated total RNA from *H*. *insolens* mycelia using Trizol reagent (Gibco) and amplified the cDNA of *Hipmo1* with a pair of oligonucleotide primers by RT-PCR using the RNA PCR Kit 3.0 instruction (Takara) (Additional file 1: Table S1). For *Hipmo1* expression, the coding region of *Hipmo1*, without the signal peptide sequence, was amplified with a primer pair containing an *Xho*I and an *Xba*I restriction site (Additional file 1: Table S1), which were synthesized based on the gene (Scyth2p4_007556) from the genomic sequencing of *H*. *insolens* (www.fungalgenomics.ca). The amplified product was enzymatically digested with *Xho*I and *Xba*I and ligated with the plasmid pPICZαA vector, linearized using *Xho*I and *Xba*I, producing the recombinant expression plasmid pPICZαA/*Hipmo1*. After it was linearized with *Pme*I, *P. pastoris* was transformed with the recombinant expression plasmid pPICZαA/*Hipmo1* by electroporation with Eppendorf Electroporator 2510 (Eppendorf Scientific). The transformants were selected on YPDS plates containing 100 mg/L of zeocin (Invitrogen).

### HiPMO1 induction and purification

HiPMO1 in transformed *P*. *pastoris* was induced and expressed according to the *Pichia* Expression System Kit Manual (Invitrogen). The culture of the transformed *P*. *pastoris* and the purification of the expressed HiPMO1 was performed according to the method previously described [29]. In brief, we purified HiPMO1 using Ni-chelating affinity chromatography on a His Trap column (GE Healthcare). For this step, 30 mM imidazole in 100 mM K_2_HPO_4_–KH_2_PO_4_ buffer (PBS buffer, pH 7.4) and 300 mM NaCl were used as a washing buffer, and 250 mM imidazole in 100 mM K_2_HPO_4_–KH_2_PO_4_ buffer (pH 7.4) and 300 mM NaCl were used as an elution buffer. Three elution fractions (about 6 mL) were obtained, pooled and dialyzed overnight at 4 °C against three changes of 10 mM HAc-NH_4_Ac buffer (pH 5.0). The purified Cu^2+^-loaded HiPMO1 was used for further functional studies.

### Protein determination, SDS-PAGE, carbohydrate staining, and N-terminal amino acid sequence analysis

The Lowry method was used for protein determination [34]. The purity of the HiPMO1 protein was determined using SDS-PAGE [35]. The HiPMO1 N-terminal amino acid sequence was determined by LC–MS/MS using a nano-LC combined with a Q Exactive mass spectrometer (Thermo Scientific) according to the method previously described [29, 36, 37]. The carbohydrates in the HiPMO1 protein were stained with the Pierce^®^ Glycoprotein Staining Kit (Thermo Scientific).

### HiPMO1 activity assay

PASC was prepared as previously described [5]. Activity assays were carried out as previously described [29]. HiPMO1 enzyme reactions on PASC occurred in 1 mL containing 5 mg/mL PASC, 5 μM Cu^2+^-loaded HiPMO1, and 1 mM Vc (ascorbate) in 10 mM ammonium acetate (pH 5.0) for 48 h at 50 °C [29]. HiPMO1 reaction products were identified using TLC, MALDI-TOF-MS/MS, LC–MS/MS, and HPAEC–PAD analysis.

### TLC and MALDI-TOF MS/MS

TLC was used to analyze the HiPMO1 products on a Silica gel 60 F254 (Merck) according to the method previously described [29]. We analyzed the HiPMO1 reaction products using MALDI-TOF-MS/MS on a 5800 MALDI-TOF/TOF analyzer (AB SCIEX), as described in previous publications [14, 29]. We showed changes in molecular mass of cello-oligosaccharides (DPn) with *m*/*z* + 16 and − 2 because of their oxidation. Types of fragmentation ions were nominated according to the described method [29, 38].

### Analysis of HtPMO1 soluble reaction products oxidized by Br_2_

We used Br_2_ to oxidize HiPMO1 reaction products using the method as described [29]. Briefly, HiPMO1 reaction products were oxidized with saturated bromine water (approximately 3%, w/v), dried under a stream of nitrogen, and then dissolved in water for MALDI-TOF MS analysis.

### LC–MS

We used two different methods of LC–MS analysis for identifying HiPMO1 reaction products. (i) Full Scan LC–MS: We analyzed HiPMO1 reaction products using Full Scan LC–MS with a HPLC analysis system and a LC–MS-2020 mass spectrometer (Shimadzu). We performed HPLC analysis on a C_18_ column (Agela Odyssil C18, 2.1 × 100 mm) with methanol and water (90:10, v/v), containing 0.2% formic acid as a mobile phase at a flow rate of 0.2 mL/min. Mass spectra were acquired in their positive and negative mode, respectively. The full scan *m*/*z* ranged from 100 to 300. (ii) SIM LC–MS/MS: We analyzed HiPMO1 reaction products using SIM LC–MS (ACQUITY UPLC and Q-TOF MS Premier, Waters) on a U3000-HPLC C18 column (Agilent Zorbax SB-C18, 150 × 4.6 mm). The SIM LC–MS settings were as follows: mobile phase A: acetonitrile (5–100%); mobile phase B: water; time: 50 min; flow rate: 0.5 mL/min; ESI mode: positive and negative ionization SIM mode; selected molecular ions: glucuronic acid (*m*/*z* 194), saccharic acid (*m*/*z* 210), and saccharic acid lactone (*m*/*z* 192). Collision-induced dissociation was used for the MS/MS analysis.

### HPAEC–PAD

We analyzed HiPMO1 reaction products hydrolyzed by beta-glucuronidase and beta-glucosidase using HPAEC–PAD as previously described [5]. The HPAEC–PAD system (ICS-3000) was equipped with a CarboPac PA200 column combination (Dionex). We annotated glucose, gluconic acid, glucuronic acid, and saccharic acid in HiPMO1 reaction products hydrolyzed by beta-glucuronidase and beta-glucosidase with the elution pattern of standard glucose, gluconic acid, glucuronic acid, and saccharic acid. The concentrations of gluconic acid, glucuronic acid, and saccharic acid in HiPMO1 reaction products hydrolyzed by beta-glucuronidase and beta-glucosidase were quantified from standard curves of gluconic acid, glucuronic acid, and saccharic acid using HPAEC–PAD analysis.

### Homology modeling

The most suitable template for modeling the HiPMO1 was found via SWISS-MODEL server (www.swissmodel.expasy.org). Sequence alignments were produced with ClustalW2 (www.ebi.ac.uk/Tools/msa/clustalw2). The final sequence alignment was submitted to SWISS-MODEL server for generating a homology model of HiPMO1. HiPMO1 model was aligned to LsAA9A:cellopentaose (PDB ID: 5NLS) [13] using PyMOL (www.pymol.org).

## Results

### Expression and purification of HiPMO1

A gene of AA9 PMOs was amplified from the thermophilic fungus *Humicola insolens* (*Scytalidium thermophilum*). We deposited the amplified AA9 gene in the GenBank (MF979101), with 4 differences in amino acids from the Scyth2p4_007556 gene of the *H*. *insolens* genome (Additional file 1: Figure S1). The gene encodes a putative AA9 PMO protein of 364 amino acids, designated as HiPMO1. A BLASTP search revealed that HiPMO1 belongs to the AA9 family of the CAZy database (CAZy) and has a catalytic domain and a carbohydrate-binding module (CBM1) [12, 26]. Using NetOGlyc 4.0 Server (www.cbs.dtu.dk/services/NetOGlyc/) and NetNGlyc 1.0 Server (www.cbs.dtu.dk/services/NetNGlyc/), we predicted 34 putative O-linked sites but no putative N-linked glycosylation sites in the deduced amino acid sequence of HiPMO1 (Additional file 1: Table S2), indicating that the HiPMO1 protein may be O-glycosylated.

**Fig. 1:**
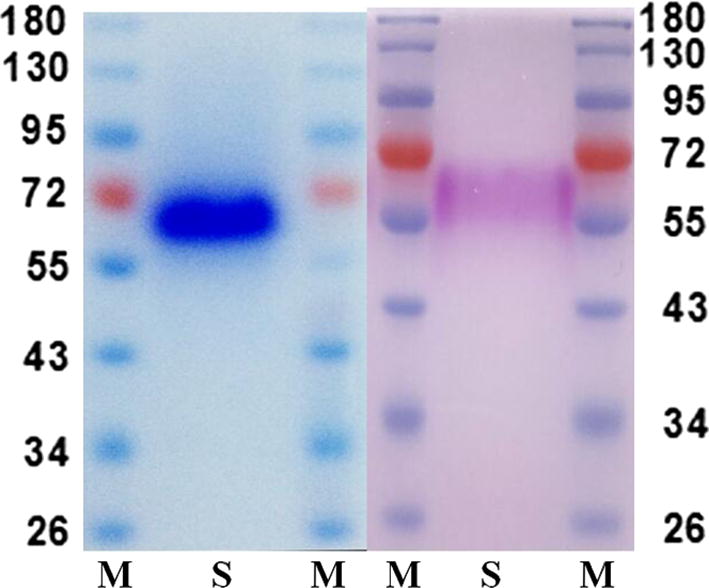
SDS-PAGE of Cu^2+^-HiPMO1 expressed in *Pichia pastoris*. HiPMO1 visualized by staining with Coomassie Brilliant Blue (left). HiPMO1 stained with Pierce^®^ Glycoprotein Staining Kit (right). Lane M, protein marker (kDa); Lane S, HiPMO1

In order to obtain the HiPMO1 protein with the N-terminal histidine residue, we used the pPICZαA vector for *Hipmo1* expression in *P*. *pastoris* [21, 29]. We purified the Cu^2+^-loaded HiPMO1 expressed in *P*. *pastoris* using nickel affinity chromatography (Fig. 1a). LC–MS/MS analysis showed that the HiPMO1 N-terminal amino acid sequence was HGHVSHIIVNGVQYR (Additional file 1: Figures S2 and S3), indicating that HiPMO1 was correctly recognized and processed in *P*. *pastoris* cells. Like other PMOs expressed in *P*. *pastoris* [22, 29, 39], the N-terminal His residue in HiPMO1 is not modified (methylated).

We predicted HiPMO1 to be a secreted protein with a potential signal peptide of 20 amino acids, MAPKTSTFLASLTGAALVAA, using SignalP (www.cbs.dtu.dk/services/SignalP/). The deduced mature protein of HiPMO1 is composed of 334 amino acids with a calculated molecular mass of 34.89979 kDa. Using SDS-PAGE, we estimated the molecular mass of the recombinant HiPMO1 protein expressed in *P*. *pastoris* to be approximately 57.5 kDa with high degree of glycosylation to 39.3%. These observations show that HiPMO1 molecular mass determined by SDS-PAGE is much higher than that estimated using the deduced amino acid sequence (34.89979 kDa), suggesting considerable glycosylation. Further periodic acid-Schiff staining showed that HiPMO1 was glycosylated (Fig. 1b), consistent with the predicted results when using the NetOGlyc 4.0 Server and SDS-PAGE analysis. Heavy glycosylation (40.4%) was also reported from PMO proteins of *Neurospora crassa* expressed in yeast [19]. Because HiPMO1 has 34 potential O-glycosylated sites located in the linker region, it is not surprising that HiPMO1 was 39.3% glycosylated in *P*. *pastoris*. Because we cannot obtain HiPMO1 native protein, the relation between the native glycosylation in *H*. *insolens* and the heavy glycosylation in *P*. *pastoris* of the enzyme is unclear. Further study on HiPMO1 glycosylation is necessary.

### HiPMO1 is able to oxidize cellulose at C1, C4, and C6 positions

**Fig. 2:**
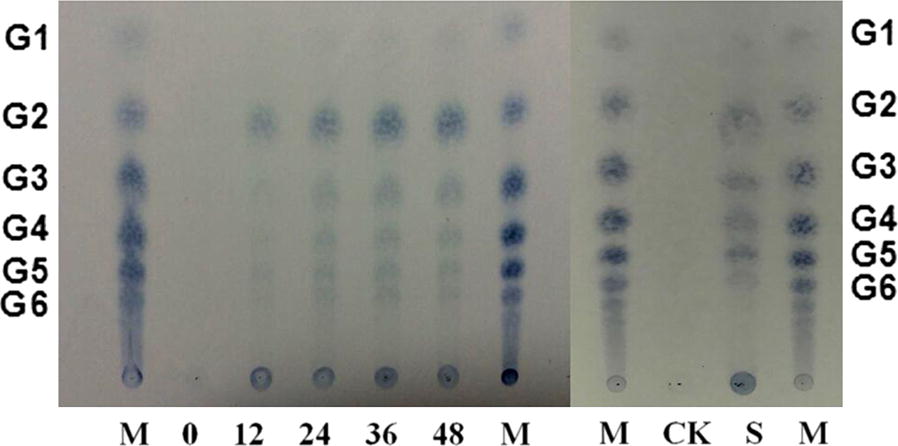
Analysis of HiPMO1 reaction products using TLC. HiPMO1 reaction for 0, 12, 24, 36, and 48 h. Lane M, standard cellulo-oligosaccharides (G1–G6); Lane S, HiPMO1 reaction products for 48 h; Lane CK, the control sample analyzed as above except without HiPMO1

**Fig. 3:**
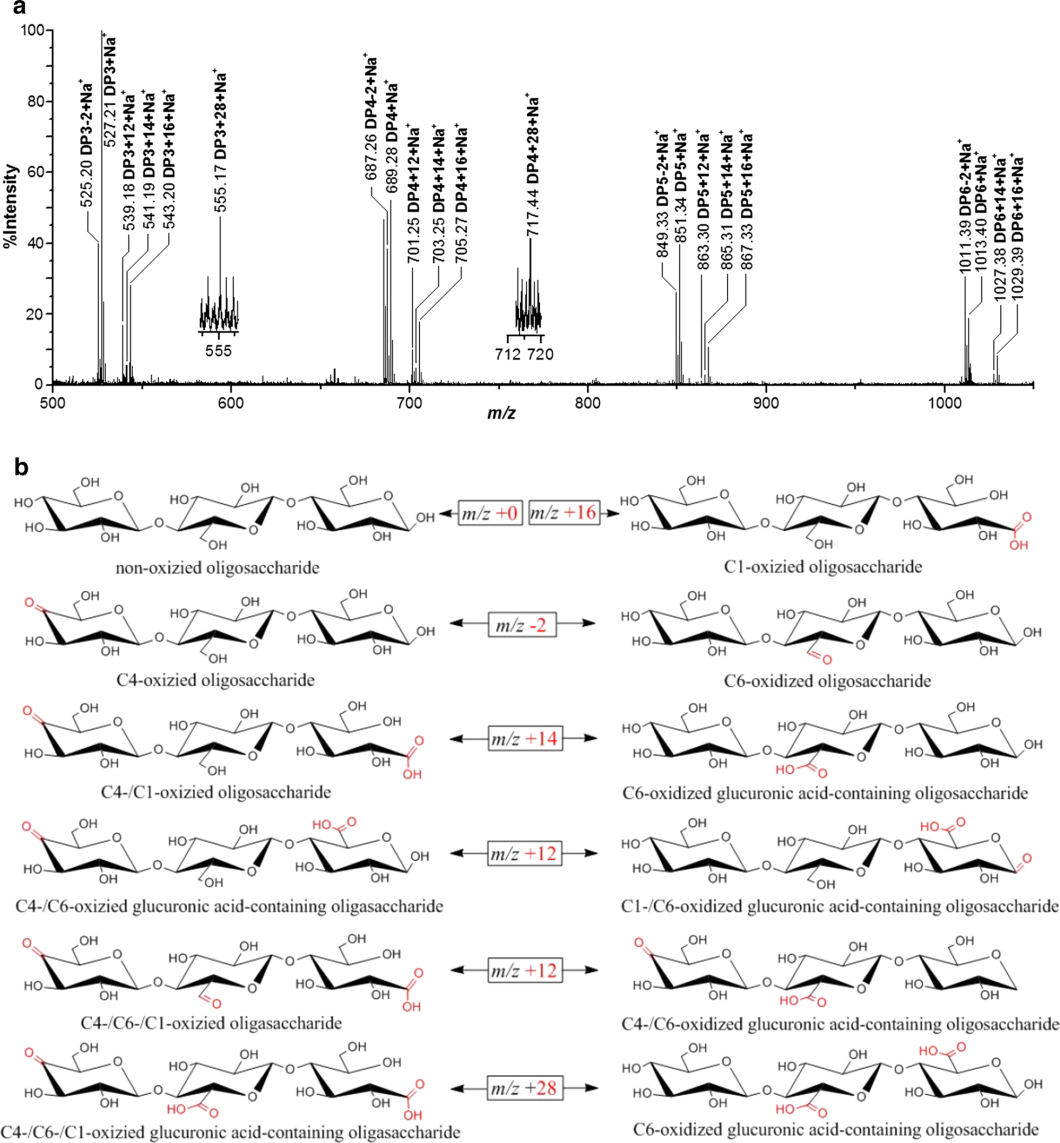
Analysis of HiPMO1 reaction products using MALDI-TOF–MS. **a** Various non-oxidized and oxidized oligosaccharides: non-oxidized oligosaccharides (*m*/*z* + 0), C4-oxidized oligosaccharides (C4-ketoaldoses) or C6-oxidized oligosaccharides (C6-hexodialdoses) (*m*/*z* − 2), C1-oxidized oligosaccharides (aldonic acids) (*m*/*z* + 16), C1-/C4-oxidized oligosaccharides (*m*/*z* + 14) or C6-oxidized glucuronic acid-containing oligosaccharides (*m*/*z* + 14), C1-/C6-oxidized glucuronic acid-containing oligosaccharide lactones or C4-/C6-oxidized glucuronic acid-containing oligosaccharides (*m*/*z* + 12), C1-/C6-/C4-oxidized glucuronic acid-containing oligosaccharides or C6-/C6-oxidized glucuronic acid-containing oligosaccharides (*m*/*z* + 28). **b** Potential structures of various oxidized and non-oxidized DP3 oligosaccharides. Only 1 of 3 potential C6-oxidized positions was marked

To determine cleavage of cellulose by HiPMO1, we analyzed reaction products of HiPMO1 using a previously established procedure [29]. The analysis of TLC showed that HiPMO1 was able to cleave cellulose to produce cell-oligosaccharides with different degrees of polymerization (DP) (Fig. 2). Further analysis of MALDI-TOF-MS showed the presence of a series of molecular ions corresponding to oxidized and non-oxidized cello-oligosaccharides (Fig. 3a). As expected, we observed C1-oxidized cello-oligosaccharides (*m*/*z* + 16) and C4- or C6-oxidized cello-oligosaccharides (*m*/*z* − 2). Unexpectedly, we also observed the special oxidized cello-oligosaccharides (*m*/*z* + 12, + 14, + 28). The observation of these special oxidized oligosaccharides (*m*/*z* + 12, + 14, + 28) shows the likely presence of C6-oxidized glucuronic acid-containing oligosaccharides and lactones (*m*/*z* + 12) of C1-/C6-oxidized glucuronic acid-containing oligosaccharides (*m*/*z* + 30) in HiPMO1 reaction products (Fig. 3b).

We further analyzed HiPMO1 reaction products using MALDI-TOF-MS/MS analysis. We selected the peak with an *m*/*z* value of 525 (*m*/*z* DP3-2)—it being the highest and corresponding to the C6- or C4-oxidized oligosaccharides—to further identify the C4 or C6 oxidized oligosaccharides. The MALDI-TOF-MS/MS analysis showed the presence of C6- or C4-oxidized fragmentation ions (Additional file 1: Figure S4 and Table S3). Notably, the MALDI-TOF-MS/MS analysis also showed the presence of the fragmentation ions oxidized only at C6, indicating that there are C6-hexodialdoses (*m*/*z* − 2) in HiPMO1 reaction products. It should be pointed out that because of low intensity of the peaks with *m*/*z* + 12, + 14 and + 28, we were not able to observe their fragmentation ions when they were analyzed using MALDI-TOF-MS/MS.

**Fig. 4:**
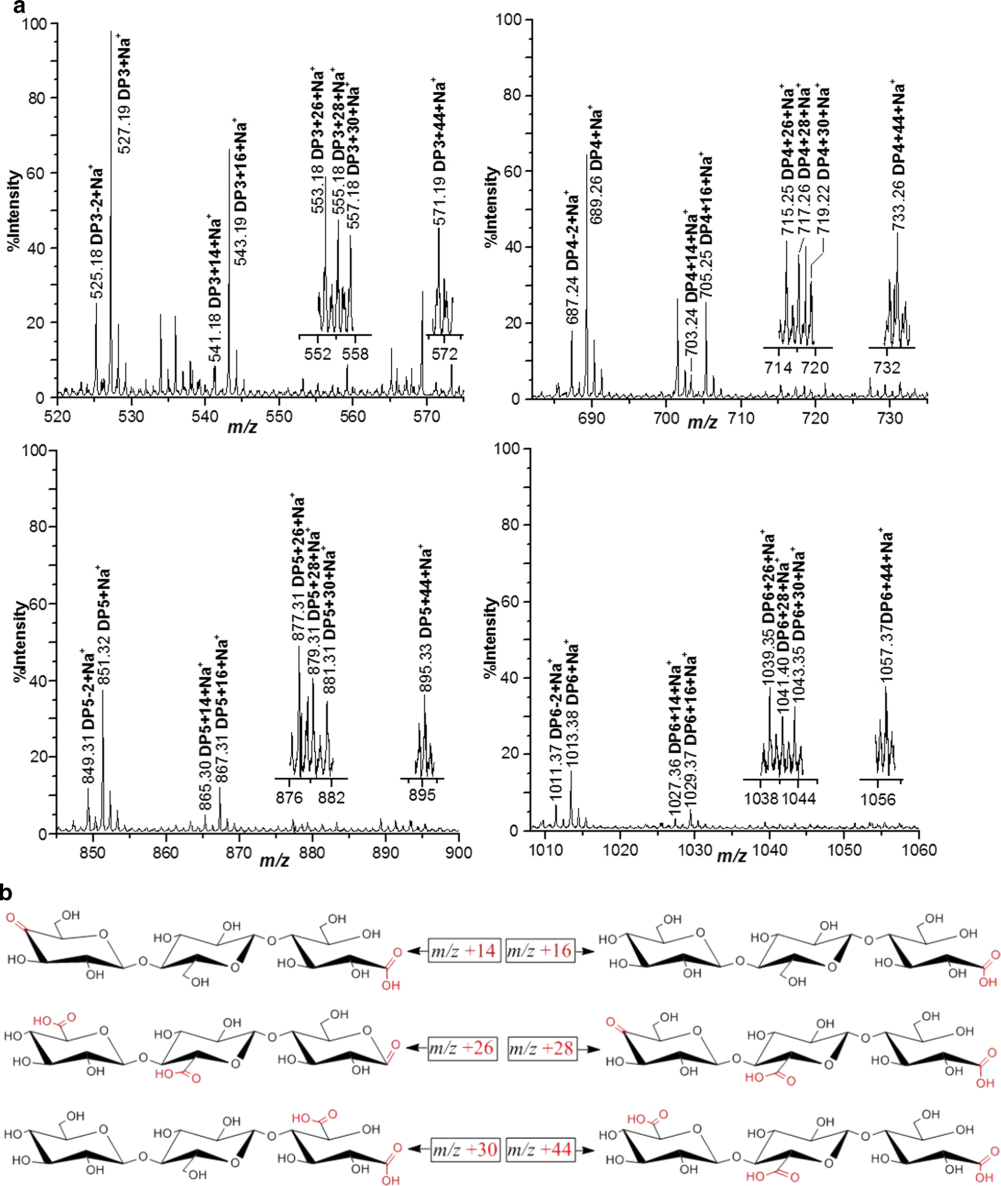
Identification of HiPMO1 reaction products after Br_2_ oxidation using MALDI-TOF–MS. **a** Various oxidized oligosaccharides: C4-/C1-oxidized oligosaccharides (*m*/*z* + 14), C1-oxidized oligosaccharides (*m*/*z* + 16), C1-/C6-/C6-oxidized glucuronic acid-containing oligosaccharides (*m*/*z* + 26), C1-/C6-/C4-oxidized glucuronic acid-containing oligosaccharides (*m*/*z* + 28), C1-/C6-oxidized glucuronic acid-containing oligosaccharides (*m*/*z* + 30), and C1-/C6-/C6-oxidized glucuronic acid-containing oligosaccharides (*m*/*z* + 44). **b** Potential structures of various oxidized DP3 oligosaccharides. Only 1 of 3 potential C6-oxidized positions was marked

To further confirm that the C4 and C6 positions were oxidized by HiPMO1, we performed the previously described chemical method wherein Br_2_ oxidizes the reaction products of PMOs [29], applying it to HiPMO1 product identification. As expected, when HiPMO1 reaction products were oxidized by Br_2_, we observed the presence of various oxidized molecular ions, such as C4-/C1-oxidized cello-oligosaccharides (*m*/*z* + 14), C1-oxidized cello-oligosaccharides (*m*/*z* + 16), C6-/C6-/C1-oxidized cello-oligosaccharides (*m*/*z* + 26), C6-/C4-/C1-oxidized cello-oligosaccharides (*m*/*z* + 28), C6-/C1-oxidized cello-oligosaccharides (*m*/*z* + 30), and C1-/C6-/C6-oxidized cello-oligosaccharides (*m*/*z* + 44) (Fig. 4), indicating the presence of C6-oxidized cello-oligosaccharides in HiPMO1 reaction products.

Together, these data have demonstrated that HiPMO1 generates products oxidized at C1, C4, and C6, similar to CtPMO1 from the thermophilic fungus *Chaetomium thermophilum* [29]. In particular, these data also showed the presence of potential new products—glucuronic acid-containing cello-oligosaccharides—in HiPMO1 reaction products.

### Determination of glucuronic acid-containing cello-oligosaccharides in HiPMO1 reaction products

**Fig. 5:**
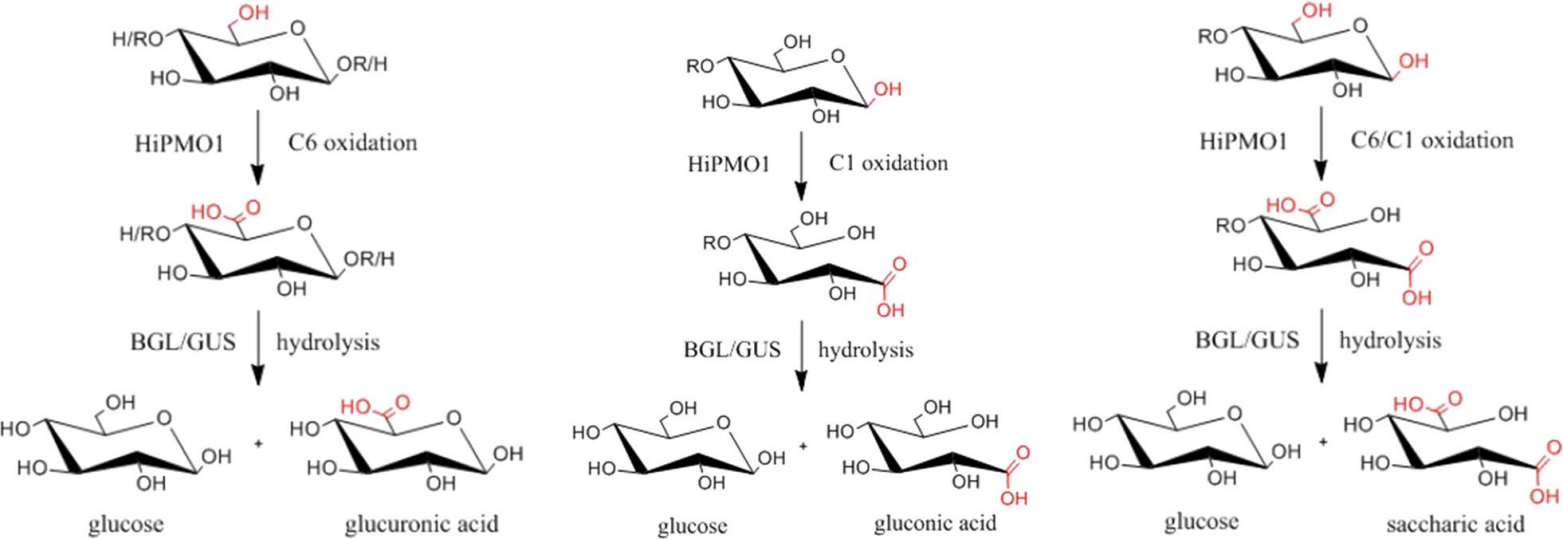
Reaction pathway for C6 and C1 oxidation of cellulose by HiPMO1 followed by hydrolyzed beta-glucuronidase and beta-glucosidase. C6 oxidation (left), C1 oxidation (middle), C6/C1 oxidation (right)

Based on MALDI-TOF-MS analysis, we observed the molecular ions at *m*/*z* + 12, + 14 in HiPMO1 reaction products, but they could not be determined as C1-/C6-oxidized glucuronic acid-containing oligosaccharides (*m*/*z* + 12) and C6-oxidized glucuronic acid-containing oligosaccharides (*m*/*z* + 14) because they have identical molecular masses with C4-/C6-oxidized oligosaccharides (*m*/*z* + 12) and C1-/C4-oxidized oligosaccharides (*m*/*z* + 14), respectively (Fig. 3b). To confirm the presence of glucuronic acid-containing cello-oligosaccharides in HiPMO1 reaction products, we used beta-glucuronidase and beta-glucosidase to hydrolyze glucuronic acid-containing cello-oligosaccharides. Because beta-glucuronidase and beta-glucosidase are exo-acting glycoside hydrolases and catalyze hydrolysis in terminal beta-d-glucuronic acid and beta-d-glucosyl residues from the non-reducing end of polysaccharides with release of glucuronic acid and glucose, respectively [40, 41], they are able to alternately hydrolyze glucuronic acid-containing cello-oligosaccharides to produce glucose, gluconic acid, glucuronic acid, and saccharic acid (Fig. 5).

**Fig. 6:**
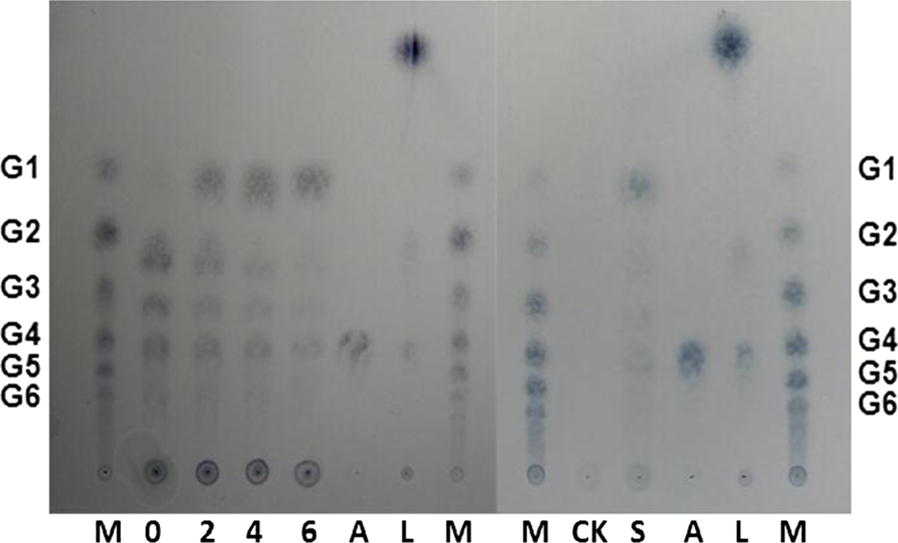
Analysis of HiPMO1 reaction products hydrolyzed by beta-glucuronidase and beta-glucosidase using TLC analysis. TLC showing HiPMO1 reaction products hydrolyzed by beta-glucuronidase and beta-glucosidase at 37 °C for 0, 2, 4, and 6 h. Lane M, standard cellulo-oligosaccharides (G1–G6); Lane S, HiPMO1 reaction products by beta-glucuronidase and beta-glucosidase at 37 °C for 6 h; Lane A, glucuronic acid; Lane L, glucuronolactone; Lane CK, the control sample, analyzed as above except without HiPMO1

**Fig. 7:**
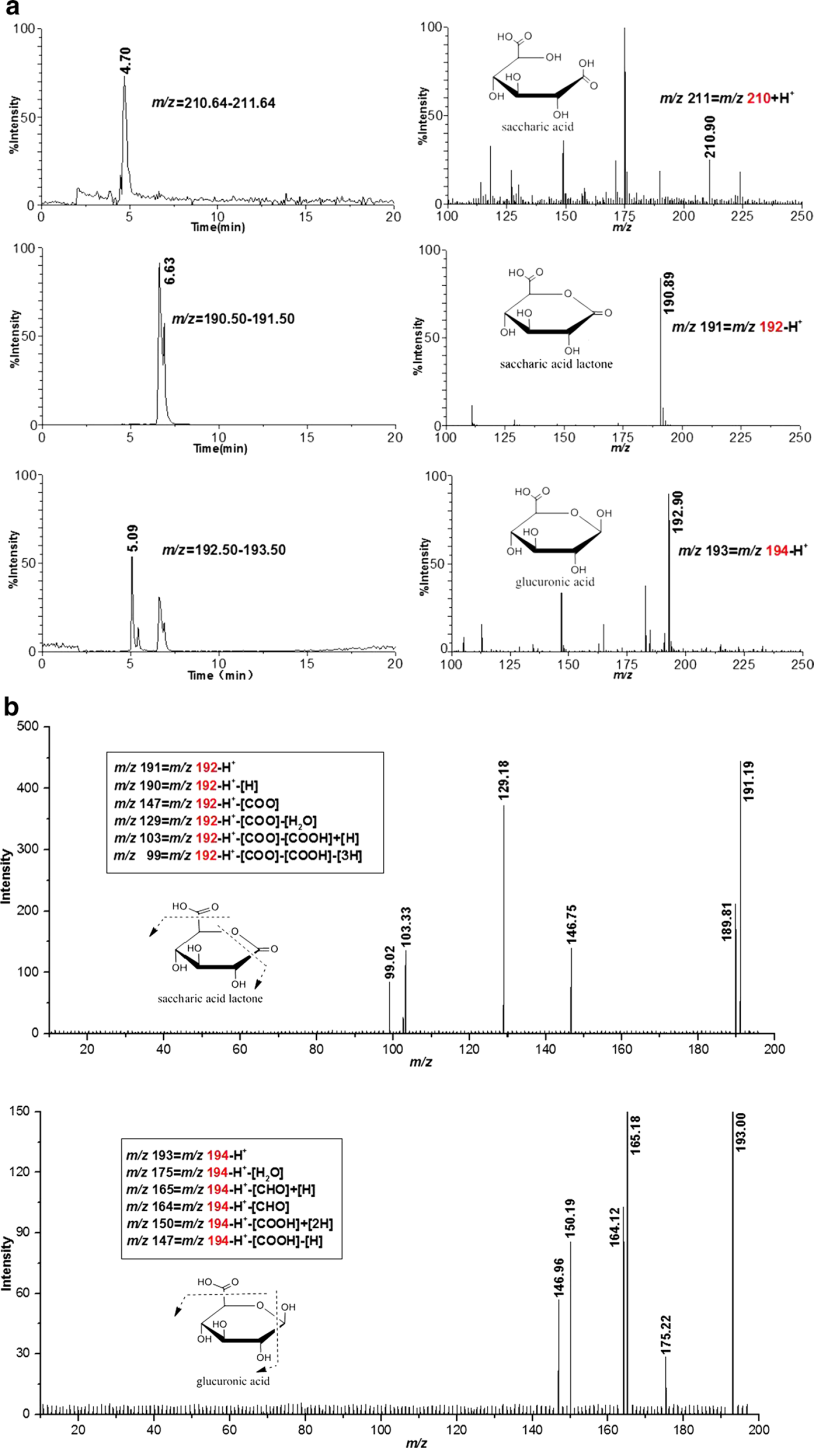
Analysis of HiPMO1 reaction products hydrolyzed by beta-glucuronidase and beta-glucosidase using SIM LC–MS/MS analysis. **a** SIM LC–MS showing extracted ion chromatograms and the corresponding mass spectra of glucuronic acid and saccharic acid (saccharic acid lactone). In positive mode: saccharic acid (*m*/*z* 210 + H^+^). In negative mode: glucuronic acid (*m*/*z* 194 − H^+^) and saccharic acid lactone (*m*/*z* 192 − H^+^). **b** The SIM MS/MS spectra showing fragmentation ions of the parent ions at *m*/*z* 192.8 for glucuronic acid (*m*/*z* 194 − H^+^) and at *m*/*z* 190.9 for saccharic acid lactone (*m*/*z* 192 − H^+^). Loss of [H] and [H_2_O] and addition of [H] is common in carbohydrate fragmentations using LC–MS/MS in the negative ion mode [38]. The loss of [H], [H_2_O], [CHO], and [COOH] and the addition of [H] from the parent ions of glucuronic acid (*m/z* 194 − H^+^) and saccharic acid lactone (*m*/*z* 192 − H^+^), generating various fragmentation ions. The MS/MS spectra of saccharic acid (*m*/*z* 210 + H^+^) were not shown, likely due to the low intensities

**Fig. 8:**
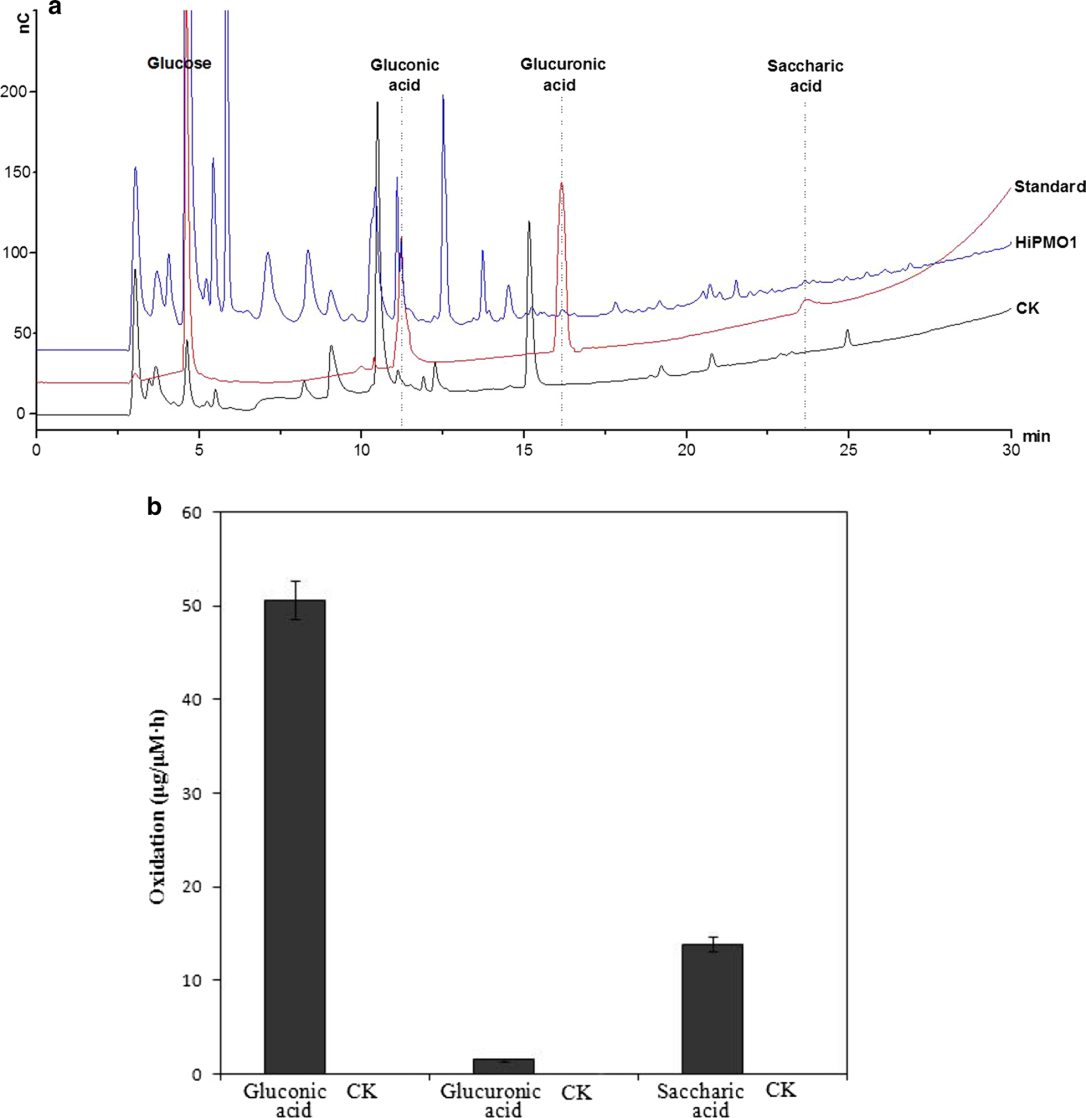
Analysis of HiPMO1 reaction products hydrolyzed by beta-glucuronidase and beta-glucosidase using HPAEC–PAD analysis. **a** Qualitative analysis: HiPMO1–HiPMO1 reaction products hydrolyzed by beta-glucuronidase and beta-glucosidase; CK-the control sample analyzed as above except without HiPMO1; standard glucose, gluconic acid, glucuronic acid, and saccharic acid. HPAEC–PAD analysis of a mixture of equal amounts of standard glucose, gluconic acid, glucuronic acid, and saccharic acid shows that saccharic acid has the lowest sensitivity, resulting in the minor peak corresponding to saccharic acid in HiPMO1 reaction products hydrolyzed by beta-glucuronidase and beta-glucosidase. **b** Quantitative analysis: the rate of formation of gluconic acid, glucuronic acid, and saccharic acid in HiPMO1 reaction products hydrolyzed with beta-glucuronidase and beta-glucosidase were quantified from standard curves of gluconic acid, glucuronic acid, and saccharic acid using HPAEC–PAD analysis. The unit of the rate was expressed in µg/µM h, the amount (µg) of product formed per enzyme molecule (µM) per unit time (h). The control sample analyzed as above except without HiPMO1 (CK)

As expected, we observed glucose, gluconic acid (gluconic acid lactone), glucuronic acid (glucuronic acid lactone), and saccharic acid (glucuronic acid lactone) in HiPMO1 reaction products hydrolyzed by beta-glucuronidase and beta-glucosidase using TLC (Fig. 6) and Full Scan LC–MS analysis in positive mode (Table 1, Additional file 1: Figure S5) and in negative mode (Table 2, Additional file 1: Figure S6). Further SIM LC–MS/MS analysis showed the presence of glucuronic acid and saccharic acid (saccharic acid lactone) (Fig. 7a, b). We further determined the presence of glucuronic acid and saccharic acid using HPAEC–PAD analysis (Fig. 8a). These data provide direct evidence of the presence of glucuronic acid-containing cello-oligosaccharides in HiPMO1 reaction products consistent with MALDI-TOF-MS analysis, indicating that HiPMO1 is able to catalyze C6 oxidation of cellulose to glucuronic acid-containing cello-oligosaccharides. In addition, the presence of saccharic acid in HiPMO1 reaction products hydrolyzed by beta-glucuronidase and beta-glucosidase indicates that HiPMO1 is able to C6-oxidize cellulose at the reducing end. Because glucuronic acid and saccharic acid are enriched by hydrolysis of glucuronic acid-containing cello-oligosaccharides with beta-glucuronidase and beta-glucosidase, we could identify glucuronic acid and saccharic acid using LC–MS and HPAEC–PAD.

**Table 1:** Positive molecular ions analysis of HiPMO1 reaction products hydrolyzed by beta-glucuronidase and beta-glucosidase

*m*/*z*	Molecular ions of potential products	*m*/*z*	Molecular ions of potential products
102	180 + Na^+^ + H^+^/2	181	180 + H^+^
103	182 + Na^+^ + H^+^/2	184	192 + 174 + 2H^+^/2
106	210 + H^+^ + H^+^/2	185	194 + 174 + 2H^+^/2
120	192 + 2H^+^ + HCOOH/2	186	196 + 174 + 2H^+^/2
122	176 + 2NH_4_^+^ + CH_3_OH/2	187	180 + 192 + 2H^+^/2
123	210 + 2NH_4_^+^/2	191	174 + 174 + 2H^+^ + CH_3_OH/2
145	194 + 2NH_4_^+^ + CH_3_COOH/2	193	192 + H^+^
149	180 + 2NH_4_^+^ + HCOOH + 2H_2_O/2	195	194 + H^+^
	194 + 2NH_4_^+^ + CH_3_OH + 2H_2_O/2	203	180 + Na^+^
152	180 + 2Na^+^ + CH_3_COOH + H_2_O/2	206	192 + 174 + 2Na^+^/2
	194 + 2Na^+^ + HCOOH + H_2_O/2	210	192 + NH_4_^+^
153	210 + 2NH_4_^+^ + CH_3_COOH/2	212	194 + NH_4_^+^
	192 + 2NH_4_^+^ + H_2_O + CH_3_COOH/2	214	196 + NH_4_^+^
158	210 + 2Na^+^ + CH_3_COOH/2	215	192 + Na^+^
	192 + 2Na^+^ + H_2_O + CH_3_COOH/2	216	180 + NH_4_^+^ + H_2_O
161	180 + 2Na^+^ + CH_3_COOH + 2H_2_O/2	217	194 + Na^+^
	194 + 2Na^+^ + HCOOH + 2H_2_O/2	228	210 + NH_4_^+^
163	196 + Na^+^ + H^+^ + CH_3_COOH + HCOOH/2	232	196 + NH_4_^+^ + H_2_O
	210 + Na^+^ + H^+^ + 2HCOOH/2	233	210 + Na^+^
166	210 + 2H^+^ + 2CH_3_COOH/2	254	176 + NH_4_^+^ + CH_3_COOH
	192 + 2H^+^ + H_2_O + 2CH_3_COOH/2	256	178 + NH_4_^+^ + CH_3_COOH
167	196 + 2Na^+^ + CH_3_COOH + CH_3_OH/2		192 + NH_4_^+^ + HCOOH
	210 + 2Na^+^ + HCOOH + CH_3_OH/2	257	196 + H^+^ + CH_3_COOH
168	180 + 2NH_4_^+^ + 2CH_3_COOH/2		210 + H^+^ + HCOOH
	194 + 2NH_4_^+^ + HCOOH + CH_3_COOH/2	264	210 + NH_4_^+^ + 2H_2_O
170	180 + 2NH_4_^+^ + 2HCOOH + CH_3_OH/2	265	210 + Na^+^ + CH_3_OH
	194 + 2NH_4_^+^ + HCOOH + 2CH_3_OH/2		196 + Na^+^ + HCOOH
173	194 + 2Na^+^ + CH_3_COOH + HCOOH/2	267	174 + H^+^ + 2HCOOH
	180 + 2Na^+^ + 2CH_3_COOH/2	272	194 + NH_4_^+^ + CH_3_COOH
174	156 + NH_4_^+^	273	194 + H^+^ + CH_3_COOH + H_2_O
175	174 + H^+^	274	196 + NH_4_^+^ + CH_3_COOH
178	180 + 174 + 2H^+^/2		210 + NH_4_^+^ + HCOOH
179	178 + H^+^	294	180 + NH_4_^+^ + CH_3_COOH + 2H_2_O
180	180 + 178 + 2H^+^/2		194 + NH_4_^+^ + HCOOH + 2H_2_O

The products were analyzed by Full Scan LC–MS in positive mode. Data are from Additional file 1: Figure S5. Because of the presence of NH_3_, CH_3_OH, HCOOH, and CH_3_COOH in the sample solutions, the products formed adduct ions with them. Glucose (*m*/*z* 180+), gluconic acid (*m*/*z* 196+), gluconic acid lactone (*m*/*z* 178+), glucuronic acid (*m*/*z* 194+), glucuronic acid lactone (*m*/*z* 176+), saccharic acid (*m*/*z* 210+), saccharic acid lactone (*m*/*z* + 192), reduced Vc (*m*/*z* 176+), oxidized Vc (*m*/*z* 174+), oxidized and dehydrate Vc (*m*/*z* 156+)

**Table 2:** Negative molecular ions analysis of HiPMO1 reaction products hydrolyzed by beta-glucuronidase and beta-glucosidase

*m*/*z*	Molecular ions of potential products	*m*/*z*	Molecular ions of potential products
102	174 − 2H^+ ^+ CH_3_OH/2	206	180 + 180 − 2H^+ ^+ 3H_2_O/2
106	178 − 2H^+ ^+ 2H_2_O/2	218	210 + 210 − 2H^+ ^+ H_2_O/2
109	174 − 2H^+ ^+ HCOOH/2	224	176 − H^+ ^+ NH_3_ + CH_3_OH
113	210 − 2H^+ ^+ H_2_O/2	227	210 − H^+ ^+ H_2_O
121	176 − 2H^+ ^+ CH_3_OH + 2H_2_O/2	230	196 − H^+ ^+ NH_3_ + H_2_O
122	178 − 2H^+ ^+ CH_3_OH + 2H_2_O/2	233	174 − H^+ ^+ CH_3_COOH
127	210 − 2H^+ ^+ HCOOH/2	236	174 − H^+ ^+ NH_3_ + HCOOH
135	194 − 2H^+ ^+ HCOOH + CH_3_OH/2	240	192 − H^+ ^+ NH_3_ + CH_3_OH
	180 − 2H^+ ^+ 2HCOOH/2		178 − H^+ ^+ NH_3_ + HCOOH
137	194 − 2H^+ ^+ HCOOH + 2H_2_O/2	243	194 − H^+ ^+ CH_3_OH + H_2_O
	180 − 2H^+ ^+ CH_3_COOH + 2H_2_O/2		180 − H^+ ^+ HCOOH + H_2_O
139	174 − 2H^+ ^+ HCOOH + CH_3_COOH/2	249	196 − H^+ ^+ 3H_2_O
141	178 − 2H^+ ^+ HCOOH + CH_3_COOH/2	252	176 − H^+ ^+ NH_3_ + CH_3_COOH
	192 − 2H^+ ^+ 2HCOOH/2	259	192 − 2H^+ ^+ Na^+ ^+ HCOOH
151	176 − 2H^+ ^+ 2H_2_O + CH_3_OH + CH_3_COOH/2		178 − 2H^+ ^+ Na^+ ^+ CH_3_COOH
	194 − 2H^+ ^+ H_2_O + CH_3_OH + CH_3_COOH/2	261	194 − 2H^+ ^+ Na^+ ^+ HCOOH
155	174 − 2H^+ ^+ H_2_O + 2CH_3_COOH/2		180 − 2H^+ ^+ Na^+ ^+ CH_3_COOH
	192 − 2H^+ ^+ 2CH_3_COOH/2	265	194 − H^+ ^+ 4H_2_O
162	194 − 2H^+ ^+ 3H_2_O + HCOOH + CH_3_OH/2	269	210 − H^+ ^+ CH_3_COOH
	180 − 2H^+ ^+ 3H_2_O + 2HCOOH/2	272	210 − H^+ ^+ NH_3_ + HCOOH
168	210 − 2H^+ ^+ 2H_2_O + CH_3_OH + CH_3_COOH/2		196 − H^+ ^+ NH_3_ + CH_3_COOH
	196 − 2H^+ ^+ 2H_2_O + HCOOH + CH_3_COOH/2	277	196 − 2H^+ ^+ Na^+ ^+ CH_3_COOH
171	194 − 2H^+ ^+ 4H_2_O + CH_3_OH + HCOOH/2		210 − 2H^+ ^+ Na^+ ^+ HCOOH
	180 − 2H^+^+4H_2_O + 2HCOOH/2	278	180 − H^+ ^+ NH_3_ + 2H_2_O + HCOOH
173	174 − H^+^		194 − H^+ ^+ NH_3_ + 2H_2_O + CH_3_O
175	176 − H^+^	281	210 − H^+ ^+ 4H_2_O
187	180 + 196 − 2H^+^/2	285	194 − H^+ ^+ CH_3_OH + CH_3_COOH
189	180 + 178 − 3H^+ ^+ Na^+^/2		180 − H^+ ^+ HCOOH + CH_3_COOH
193	194 − H^+^	289	194 − H^+ ^+ 2H_2_O + CH_3_COOH
195	196 − H^+^		180 − H^+ ^+ H_2_O + CH_3_OH + CH_3_COOH
196	180 − H^+ ^+ NH_3_	291	196 − H^+ ^+ 2H_2_O + CH_3_COOH
197	180 − H^+ ^+ H_2_O		210 − H^+ ^+ 2H_2_O + HCOOH
200	210 + 192 − 2H^+^/2	292	180 − H^+ ^+ NH_3_ + 2H_2_O + CH_3_COOH
204	196 + 196 − 2H^+ ^+ H_2_O/2 180 + 194 − 2H^+ ^+ 2H_2_O/2		194 − H^+ ^+ NH_3_ + 2H_2_O + HCOOH

The products were analyzed by Full Scan LC–MS in negative mode. Data are from Additional file 1: Figure S6. Because of the presence of NH_3_, CH_3_OH, HCOOH, and CH_3_COOH in the sample solutions, the products formed adduct ions with them. Glucose (*m*/*z* 180−), glucuronic acid (*m*/*z* 194−), gluconic acid (*m*/*z* 196−), saccharic acid (*m*/*z* 210−), gluconic acid lactone (*m*/*z* 178−), saccharic acid lactone (*m*/*z* 192−), reduced Vc (*m*/*z* 176−), oxidized Vc (*m*/*z* 174−)

To evaluate the different oxidation activities of HiPMO1, we carried out a quantitative analysis of C1-oxidized gluconic acid, C6-oxidized glucuronic acid, and C1-/C6-oxidized saccharic acid in HiPMO1 reaction products hydrolyzed by beta-glucuronidase and beta-glucosidase using HPAEC–PAD. The rate of formation of these oxidized products is shown in Fig. 8b, indicating that the rate of C1 and C6 oxidation of HiPMO1 is different. Glucuronic acid has a low yield possibly because HiPMO1 may C6-oxidize cellulose mainly at the reducing end, resulting in the formation of saccharic acid. Because of no standard C4-oxidized products available, we cannot quantitatively determine C4-oxidized products. In addition, it is difficult to compare oxidative rate of different PMOs since activity assays were carried out with varying substrates under highly varying conditions, especially producing complex products.

C1-/C6-oxidized glucuronic acid-containing cello-oligosaccharides (*m*/*z* + 30) are hydrolyzed by beta-glucuronidase and beta-glucosidase to yield saccharic acid, but we could not observe C1-/C6-oxidized glucuronic acid-containing cello-oligosaccharides (*m*/*z* + 30) using MALDI-TOF-MS analysis. There are two possible reasons for this. One is that C1-/C6-oxidized glucuronic acid-containing cello-oligosaccharides (*m*/*z* + 30) may exist mainly in the form of lactones (*m*/*z* + 12), which were hydrolyzed by beta-glucuronidase and beta-glucosidase to yield saccharic acid and saccharic acid lactose. These C1-/C6-oxidized glucuronic acid-containing cello-oligosaccharide lactones (*m*/*z* + 12) were obviously observed using MALDI-TOF-MS analysis (Fig. 3b). Furthermore, LC–MS/MS analysis and HPAEC–PAD analysis also support the existence of these lactones (Tables 1, 2, Figs. 7, 8). The other is that a minor amount of C1-/C6-oxidized glucuronic acid-containing cello-oligosaccharides (*m*/*z* + 30) cannot be detected by MALDI-TOF-MS analysis, even though they exist in HiPMO1 reaction products. Because oligosaccharides are highly hydrophilic and lack basic groups, they have low ionization efficiency [42, 43]. The low ionization efficiency likely results in not being able to detect a minor amount of oligosaccharides using MALDI-TOF-MS analysis. Unsurprisingly, there were a lot of molecular ions in Full Scan LC–MS analysis because of diverse combinations of three positive ions (H^+^, +Na^+^, and NH_4_^+^), four solvents (H_2_O, HCOOH, CH_3_COOH, and CH_3_OH), three forms of Vc (reduced Vc, oxidized Vc, and oxidized and dehydrate Vc), and seven kinds of monosaccharides (glucose, gluconic acid, gluconic acid lactone, glucuronic acids, glucuronic acid lactone, saccharic acid, and saccharic acid lactone) in different types and numbers. Because LC–MS analysis was performed under acidic conditions, gluconic acid, glucuronic acids, and saccharic acids are in equilibrium with their intramolecular esters (lactones), including gluconic acid lactone, glucuronic acid lactone, and saccharic acid lactone. On the contrary, because we carried out HPAEC–PAD analysis under basic conditions, gluconic acid, glucuronic acid, and saccharic acid were entirely in the form of free acids and not in the form of lactones.

Combined with the MALDI-TOF-MS analysis, the TLC, LC–MS/MS, and HPAEC–PAD analysis results determined the presence of glucuronic acid-containing cello-oligosaccharides in HiPMO1 reaction products. Also, we showed that a C1-/C4-/C6-oxidizing PMO (CtPMO1) from *Chaetomium thermophilum* [29] was able to oxidize cellulose to form glucuronic acid-containing cello-oligosaccharides (Additional file 1: Figures S7, S8a, b), similar to HiPMO1. This is the first observation of glucuronic acid-containing cello-oligosaccharides in PMO reaction products.

### Structural model of HiPMO1

Using SWISS-MODEL, we obtained the homology model of HiPMO1 with *Thermoascus aurantiacus* TaGH61A (PDB id 2YET) as a template [10]. HiPMO1 shared a high identity of 53.33% with the template TaGH61A (Additional file 1: Figure S9). The homology model showed that three highly conserved amino acid residues essential for catalysis, His1, His87, and Tyr173, were clustered at the flat substrate-binding surface near the N-terminus of HiPMO1, and five aromatic residues possibly involved in HiPMO1-substrate interactions, Tyr24, Phe43, Trp83, His162, and Trp210, were present on the flat substrate-binding surface of HiPMO1 (Additional file 1: Figure S10). In addition, a conserved Ser86 residue, which is immediately before the copper active site His87 ligand, was present on the flat substrate-binding surface of HiPMO1 and it was suggested to contribute to interactions with cellulose [13, 23]. Further docking study that HiPMO1 model was aligned to LsAA9A:cellopentaose (PDB ID: 5NLS) [13] using PyMOL showed that the active site of HiPMO1 was close to hydrogen of C6 carbon of the soluble cellopentaose substrate (Additional file 1: Figure S11). These data support C6 oxidation of HiPMO1 to cellulose or cello-oligosaccharides.

## Discussion

Based on MALDI-TOF-MS analysis, the presence of C6-hexodialdoses in reaction products of *Thermoascus aurantiacus* GH61A and *Podospora anserina* GH61B have been suggested [10, 21]. Using Br_2_ oxidation and MALDI-TOF-MS analysis, our previous studies clearly showed that CtPMO1 from *Chaetomium thermophilum* generated products oxidized at C6 (C6-hexodialdoses) [29]. In the present study, we demonstrate, for the first time, that HiPMO1 is able to C6-oxidize cellulose to form glucuronic acid-containing cello-oligosaccharides.

Our study provides a new mechanism of cellulose degradation by C6 oxidation with polysaccharide monooxygenase followed by hydrolysis with beta-glucuronidase and beta-glucosidase, which differs from the mechanism of cellulose degradation by cleavage of C1 and C4 oxidation with polysaccharide monooxygenase [5, 17, 23, 26, 27]. This new mechanism of cellulose degradation may have possible biological implications. HiPMO1 C6-oxidized reaction products—glucuronic acid-containing cello-oligosaccharides—may be hydrolyzed by beta-glucuronidase and beta-glucosidase to produce glucose, glucuronic acid, and saccharic acid.

Unlike glucose as an energy source in most organisms, glucuronic acid and saccharic acid may play other biological, physiological, and ecological roles. Glucuronic acid and saccharic acid, as important organic acids, may act as chelates to stimulate manganese peroxidase activity for lignin depolymerization as oxalic acid [44]. Also, they may chelates toxic heavy metal ions in the environment for protecting living organisms. In cells, glucuronic acid may be further metabolized to highly reactive metabolites, such as vitamin C and xylulose, via the uronic acid pathway [45, 46]. Scientists have reported that saccharic acid, as an organic acid, plays important physiological roles in regulating hormones, increasing the immune function, and reducing the risks of cancer [47–49].

In the present study, we observed saccharic acid in HiPMO1 reaction products followed by hydrolysis with beta-glucuronidase and beta-glucosidase. To our knowledge, this is a new enzymatic pathway for the formation of saccharic acid, differing from the uronic acid pathway in which at least 10 enzymes catalyze the formation of saccharic acid [50]. It also differs from the synthetic glucaric acid pathway in which genic engineering strains are constructed by co-expressing the genes encoding myo-inositol oxygenase and uronate dehydrogenase to produce saccharic acid in *E*. *coli*, *Saccharomyces cerevisiae*, and *Pichia pastoris* [51–53]. Our present enzymatic pathway of saccharic acid formation by PMOs is simple, and it may provide a new way for production of saccharic acid.

It should be pointed out that the glycosidic bond of glucuronic acid-containing cello-oligosaccharides may also be cleaved at the C4-position by polysaccharide lyase (PL), belonging to the PL20 family, via a beta-elimination mechanism to produce a reducing end on one fragment of cello-oligosaccharides and an unsaturated ring on the non-reducing end of the second fragment of cello-oligosaccharides [46, 54, 55]. It was reported that when the thermophilic fungus *Myceliophthora thermophila* was grown on biomass, a PL belonging to the PL20 family was up-regulated and expressed based on data from transcriptome and secretome. This suggested that the enzyme might be involved in biomass degradation [32, 56]. Therefore, we postulate that PMOs might be able to cleave cellulose via C6 oxidation with PL20.

It is unclear how HiPMO1 catalyzes the formation of glucuronic acid-containing cello-oligosaccharides. It has been reported that galactose oxidase, belonging to the AA5_2 subfamily, has the ability to oxidize the hydroxyl group at the C-6 position of sugar to aldehyde and uronic acid. Galactose oxidase was reported not only to convert the primary alcohol group at C-6 of galactose to an aldehyde but also to a carboxylic acid (ROH → RCHO → RCOOH) [57, 58]. Recently, it was shown that 2 novel alcohol oxidases (AlcOx), belonging to the AA5_2 subfamily, catalyzed the oxidation of diverse alcohols to the corresponding aldehydes with H_2_O_2_ production [59]. Even more recently, a raffinose oxidase in the AA5_2 subfamily from *Colletotrichum graminicola* was found to have the ability to C6-oxidize galactose to an aldehyde and the corresponding uronic acid, with its hydrate as the intermediate (ROH → RCHO → RCH(OH)_2_ → RCOOH) [60]. Thanks to in-depth research, galactose oxidase has been found to be the best-characterized member of the AA5 family [61–63]. The accepted mechanism of C6 oxidation of galactose oxidase is called the ping-pong mechanism, consisting of 4 stages (the oxidation of substrate, the release of oxidized substrate, the formation of superoxide, and the release of H_2_O_2_) (RCH_2_OH + O_2_ → RCHO + H_2_O_2_) [63]. Because PMO and galactose oxidase belong to copper-dependent metalloenzymes and have similar active sites (Additional file 1: Figure S12a), HiPMO1 might have a similar mechanism to galactose oxidase when catalyzing cellulose to form glucuronic acid-containing cello-oligosaccharides as well as H_2_O_2_ (Additional file 1: Figure S12b and c) [59, 63]. Further study for this is necessary in the future.

It should be noted that glyoxal oxidase, belonging to the AA5_1 subfamily, with a broad substrate specificity for the oxidation of aldehydes to the corresponding carboxylic acids [64], was also reported to convert glycerol to glyceric acid with glyceraldehyde as the intermediate (ROH + O_2_ → RCHO + H_2_O_2_; RCHO + O_2_ → RCOOH + H_2_O_2_) [65, 66]. These pieces of evidence indicate that it might be common for the hydroxyl group of substrates (sugar and alcohols) to be oxidized to the corresponding aldehyde and carboxyl group in the AA group, especially in the AA5 family.

HiPMO1 can oxidize cellulose at C1, C4, and C6. The evidence that there are C1-/C6-oxidized and C4-/C6-oxidized cello-oligosaccharides in HiPMO1 reaction products suggests that C6 oxidation of HiPMO1 is likely related to its C1 and C4 oxidation. Recently, it was reported that oxidative cleavage of cellulose by a PMO from *Streptomyces coelicolor* (ScLPMO10C) depends on H_2_O_2_ (R-H + H_2_O_2_ → ROH + H_2_O) [67, 68]. Based on the dependence of H_2_O_2_, it is possible that HiPMO1 might use H_2_O_2_, which might be produced via C6 oxidation of cellulose by HiPMO1, to cleave cellulose via C1 and C4 oxidation.

## Conclusion

**Fig. 9:**
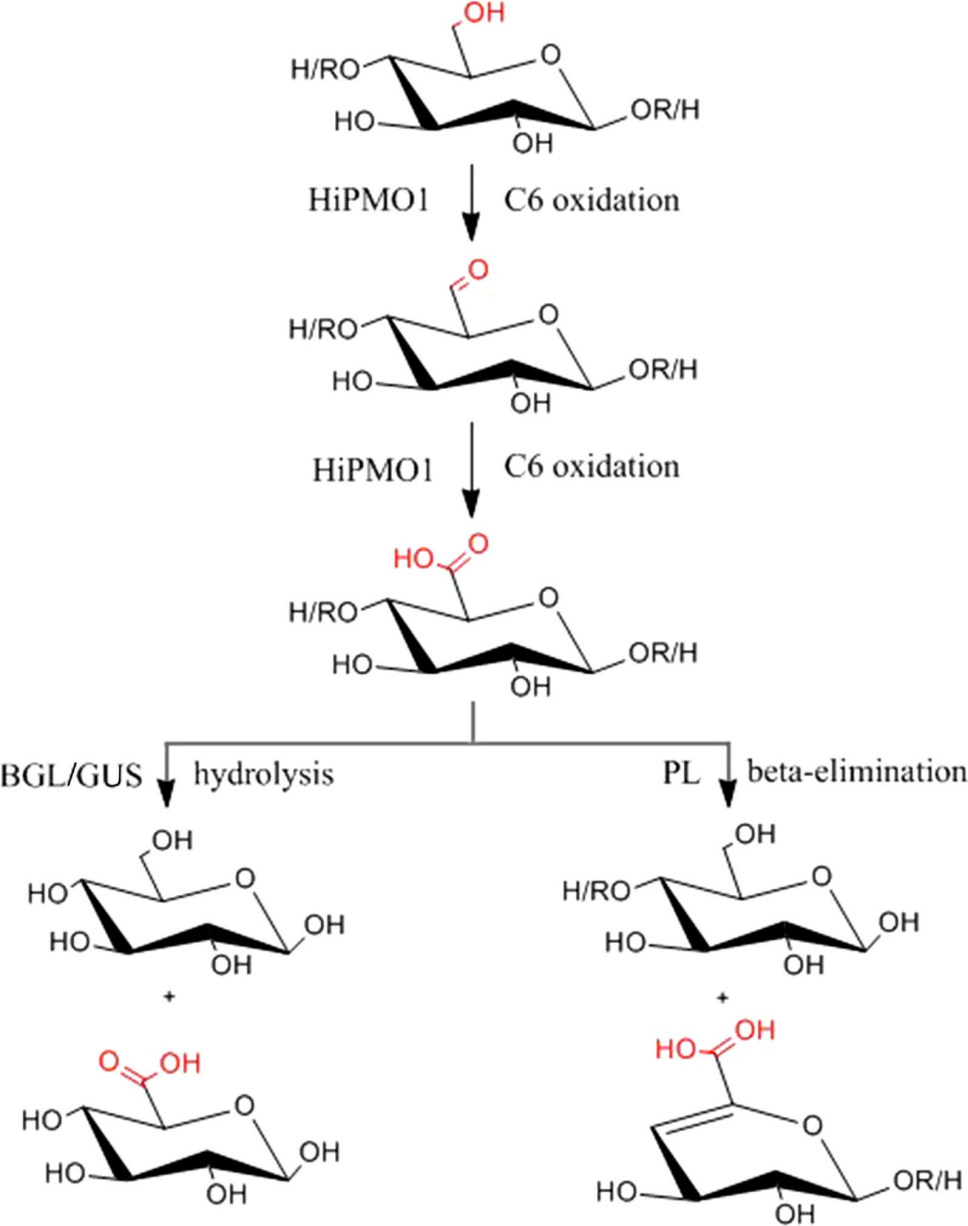
Proposed conversion of cellulose to glucuronic acid-containing cello-oligosaccharides by HiPMO1. HiPMO1 oxidizes the primary alcohol group at C-6 of cellulose to the aldehyde group and the carboxylic group (ROH → RCHO → RCOOH). The resulting glucuronic acid-containing cello-oligosaccharides might be further cleaved via hydrolysis by beta-glucuronidase (GUS) and beta-glucosidase (BGL) to yield glucose, glucuronic acid, and saccharic acid, and cleaved via beta-elimination by PL to produce unsaturated cello-oligosaccharides

HiPMO1 oxidizes C6 of cellulose to form glucuronic acid-containing cello-oligosaccharides followed by hydrolysis with beta-glucosidase and beta-glucuronidase to yield glucose, glucuronic acid, and saccharic acid, and even possibly by beta-eliminative cleavage to produce unsaturated cello-oligosaccharides. Based on our present data and knowledge, we propose a possible reaction pathway of cellulose to glucuronic acid-containing cello-oligosaccharides by HiPMO1 (Fig. 9). This study provides a new mechanism for cellulose cleavage by C6 oxidation of HiPMO1.

## Additional file

**Additional file 1: Figure S1.** Sequence alignment of HiPMO1 (MF979101) from *Humicola insolens* (*Scytalidium thermophilum*) CGMCC3.18482 and PMO (Scyth2p4_007556) from *Scytalidium thermophilum* CBS 625.91 using ClastalW2. The MF979101 gene has 4 amino acid differences to the Scyth2p4_007556 gene. **Figure S2.** HiPMO1 N-terminal amino acid sequence analysis using LC-MS. LC-MS analysis of the digested CtPMO1 protein with trypsin reveals a peak *m*/*z* of 572.6396. The *m/z* value is 1/3 of the molecular weight of the peptide HGHVSHIIVNGVQYR, indicating that the *m/z* 572.6396 ion is triply charged (*z* = 3). The extracted-ion chromatogram (XIC) of the peptide HGHVSHIIVNGVQYR was also shown. **Figure S3.** HiPMO1 N-terminal amino acid sequence analysis using LC-MS/MS. LC-MS/MS analysis shows that fragmentation *m/z* values of the *m/z* 572.6408 ion agree with the molecular weight of the corresponding fragmentations of the peptide HGHVSHIIVNGVQYR. These data indicate that the N-terminal amino acid sequence of HiPMO1 is HGHVSHIIVNGVQYR. **Figure S4.** The analysis of MALDI-TOF-MS/MS of HiPMO1 reaction products. We selected the highest peak at *m/z* 525 from MALDI-TOF-MS analysis for MS/MS. MS/MS data were acquired on the mass *m/z* range of 100–550. We observed the various fragmentation ions of the main HiPMO1 C4 or C6 oxidized product (*m/z* 525). **Figure S5.** Molecular ion peaks of HiPMO1 reaction products hydrolyzed by beta-glucuronidase and beta-glucosidase by Full Scan LC-MS in positive mode. Most molecular ion peaks have an absolute intensity exceeding 1,000. **Figure S6.** Molecular ion peaks of HiPMO1 reaction products hydrolyzed by beta-glucuronidase and beta-glucosidase by Full Scan LC-MS in negative mode. Most molecular ion peaks have an absolute intensity exceeding 200. **Figure S7.** Analysis of CtPMO1 reaction products hydrolyzed by beta-glucuronidase and beta-glucosidase using SIM LC-MS/MS analysis. SIM LC-MS showing extracted ion chromatograms and the corresponding mass spectra of glucuronic acid and saccharic acid (saccharic acid lactone). In positive mode: saccharic acid (*m/z* 210+H^+^), saccharic acid lactone (*m/z* 192+H^+^) and glucuronic acid (*m/z* 194+H^+^). **Figure S8.** Analysis of CtPMO1 reaction products hydrolyzed by beta-glucuronidase and beta-glucosidase using HPAEC-PAD analysis. (**a**) Qualitative analysis: CtPMO1-CtPMO1 reaction products hydrolyzed by beta-glucuronidase and beta-glucosidase; CK-the control sample analyzed as above except without CtPMO1; Standard-glucose, gluconic acid, glucuronic acid, and saccharic acid. (**b**) Quantitative analysis: The rate of formation of gluconic acid, glucuronic acid and saccharic acid in CtPMO1 reaction products hydrolyzed with beta-glucuronidase and beta-glucosidase were quantified from standard curves of gluconic acid, glucuronic acid and saccharic acid using HPAEC-PAD analysis. The unit of rate was expressed in µg/µM h, the amount (µg) of product formed per enzyme molecule (µM) per unit time (h). The control sample analyzed as above except without CtPMO1 (CK). **Figure S9.** Sequence alignment of catalytic domains of HiPMO1 and *Thermoascus aurantiacus* TaGH61 (PDB ID: 2YET) using ClastalW2. **Figure S10.** Homology model of the catalytic domain of HiPMO1 with *Thermoascus aurantiacus* TaGH61 (PDB ID: 2YET) as a template using SWISS-MODEL. The globally conserved residues adjacent to the copper are colored in red. The aromatic and Ser86 residues are colored in blue. The copper ion is shown as an orange sphere. **Figure S11.** Docking study of HiPMO1 binding with cellopentaose using PyMOL. HiPMO1 model was aligned to LsAA9A:cellopentaose (PDB ID: 5NLS). The globally conserved residues adjacent to the copper are colored in red. The aromatic and Ser86 residues are colored in blue. The copper ion is shown as an orange sphere. The carbon atoms of cellopentaose are colored in green, and oxygen atoms of cellopentaose are colored in light red. **Figure S12.** Speculative mechanism of C6 oxidation by HiPMO1. (**a**) Active sites of galactose oxidase (GO) and HiPMO1 [59, 63]. Four ligand atoms directly coordinating copper were shown. C6 oxygen atom of substrate (S) coordinating copper was also shown. (**b**) The mechanism of C6 oxidation by galactose oxidase (GO) [59, 63]. (**c**) Speculative mechanism of C6 oxidation by HiPMO1. In the presence of reductants (electron donors), HiPMO1-Cu(II)-H1 is first reduced to HiPMO1-Cu(I)-H1 (priming reduction), which reacts with O_2_ in the presence of substrate, resulting to generation of H_2_O_2_ and HiPMO1-Cu(II)-H1•. The resulting HiPMO1-Cu(II)-H1• reacts with substrate, leading to generation of RCHO or RCOOH. H1• denotes the His1 radical cofactor of HiPMO1, similar to the Tyr272 radical cofactor of galactose oxidase (GO)(Y272•). **Table S1.** List of primers used for PCR of the *Hipmo1* gene. The pair of oligonucleotide primers (D1 and D2) was synthesized based on the gene (Scyth2p4_007556) from the genomic sequencing of *H*. *insolens* (www.fungalgenomics.ca). **Table S2**. The putative potential O-linked glycosylation sites of HiPMO1. A list of potential glycosylation sites showed their positions in the sequence and the prediction confidence scores. Only the sites with scores higher than 0.5 are predicted to be glycosylated. **Table S3.** The analysis of fragmentation ions of the peak of DP_3_-2 (*m/z* 525) of HiPMO1 reaction products according to Additional file 1: Figure S4. Table S3 shows the type of fragmentation ions and the potential oxidized positions. Fragmentation ion types were nominated according to the methods previously described [29, 38].

## Abbreviations

AA: auxiliary activity
DP: degree of polymerization
HiPMO1: a PMO from *Humicola insolens*
HPAEC–PAD: high-performance anion exchange chromatography with pulsed amperometric detection
LC–MS: liquid chromatography–mass spectrometry
LC–MS/MS: liquid chromatography–tandem mass spectrometry
MALDI-TOF-MS/MS: matrix-assisted laser desorption/ionization-time-of-flight tandem mass spectrometry
PASC: phosphoric acid-swollen cellulose
PCR: polymerase chain reaction
PMOs: polysaccharide monooxygenases
SDS-PAGE: sodium dodecyl sulfate polyacrylamide gel electrophoresis
TLC: thin-layer chromatography
SIM: selected-ion-monitoring
