# Supplementary material for: Polysaccharide monooxygenase-catalyzed oxidation of cellulose to glucuronic acid-containing cello-oligosaccharides
Source: Biotechnol Biofuels. 2019 Feb 27;12:42. doi: 10.1186/s13068-019-1384-0 (PMC6391835; doi:10.1186/s13068-019-1384-0)
Supplement: Supplementary file 1 — Additional file 1: Figure S1. Sequence alignment of HiPMO1 (MF979101) from Humicola insolens (Scytalidium thermophilum) CGMCC3.18482 and PMO (Scyth2p4_007556) from Scytalidium thermophilum CBS 625.91 using ClastalW2. The MF979101 gene has 4 amino acid differences to the Scyth2p4_007556 gene. Figure S2. HiPMO1 N-terminal amino acid sequence analysis using LC-MS. LC-MS analysis of the digested CtPMO1 protein with trypsin reveals a peak m/z of 572.6396. The m/z value is 1/3 of the molecular weight of the peptide HGHVSHIIVNGVQYR, indicating that the m/z 572.6396 ion is triply charged (z = 3). The extracted-ion chromatogram (XIC) of the peptide HGHVSHIIVNGVQYR was also shown. Figure S3. HiPMO1 N-terminal amino acid sequence analysis using LC-MS/MS. LC-MS/MS analysis shows that fragmentation m/z values of the m/z 572.6408 ion agree with the molecular weight of the corresponding fragmentations of the peptide HGHVSHIIVNGVQYR. These data indicate that the N-terminal amino acid sequence of HiPMO1 is HGHVSHIIVNGVQYR. Figure S4. The analysis of MALDI-TOF-MS/MS of HiPMO1 reaction products. We selected the highest peak at m/z 525 from MALDI-TOF-MS analysis for MS/MS. MS/MS data were acquired on the mass m/z range of 100–550. We observed the various fragmentation ions of the main HiPMO1 C4 or C6 oxidized product (m/z 525). Figure S5. Molecular ion peaks of HiPMO1 reaction products hydrolyzed by beta-glucuronidase and beta-glucosidase by Full Scan LC-MS in positive mode. Most molecular ion peaks have an absolute intensity exceeding 1,000. Figure S6. Molecular ion peaks of HiPMO1 reaction products hydrolyzed by beta-glucuronidase and beta-glucosidase by Full Scan LC-MS in negative mode. Most molecular ion peaks have an absolute intensity exceeding 200. Figure S7. Analysis of CtPMO1 reaction products hydrolyzed by beta-glucuronidase and beta-glucosidase using SIM LC-MS/MS analysis. SIM LC-MS showing extracted ion chromatograms and the corresponding mass spectra of glucuronic acid and [file 13068_2019_1384_MOESM1_ESM.doc]

**Polysaccharide monooxygenase-catalyzed oxidation of cellulose to glucuronic acid-containing cello-oligosaccharides**

Jinyin Chen, Xiuna Guo, Min Zhu, Chen Chen, Duochuan Li*

Department of Mycology, Shandong Agricultural University, Taian, Shandong 271018, China

*Corresponding author. Tel: 86-538-8249071, Fax: 86-538-8226399, E-mail: lidc20@163.com

**Additional files**

**Additional file 1:**

**Figure S1.** Sequence alignment of HiPMO1 (MF979101) from *Humicola insolens* (*Scytalidium thermophilum*) CGMCC3.18482 and PMO ([Scyth2p4_007556](http://genome.fungalgenomics.ca/new_gene_model_pages/gene_model_page.php?gmid=Scyth2p4_007556)) from *Scytalidium thermophilum* CBS 625.91 using ClastalW2. The MF979101 gene has 4 amino acid differences to the [Scyth2p4_007556](http://genome.fungalgenomics.ca/new_gene_model_pages/gene_model_page.php?gmid=Scyth2p4_007556)gene.


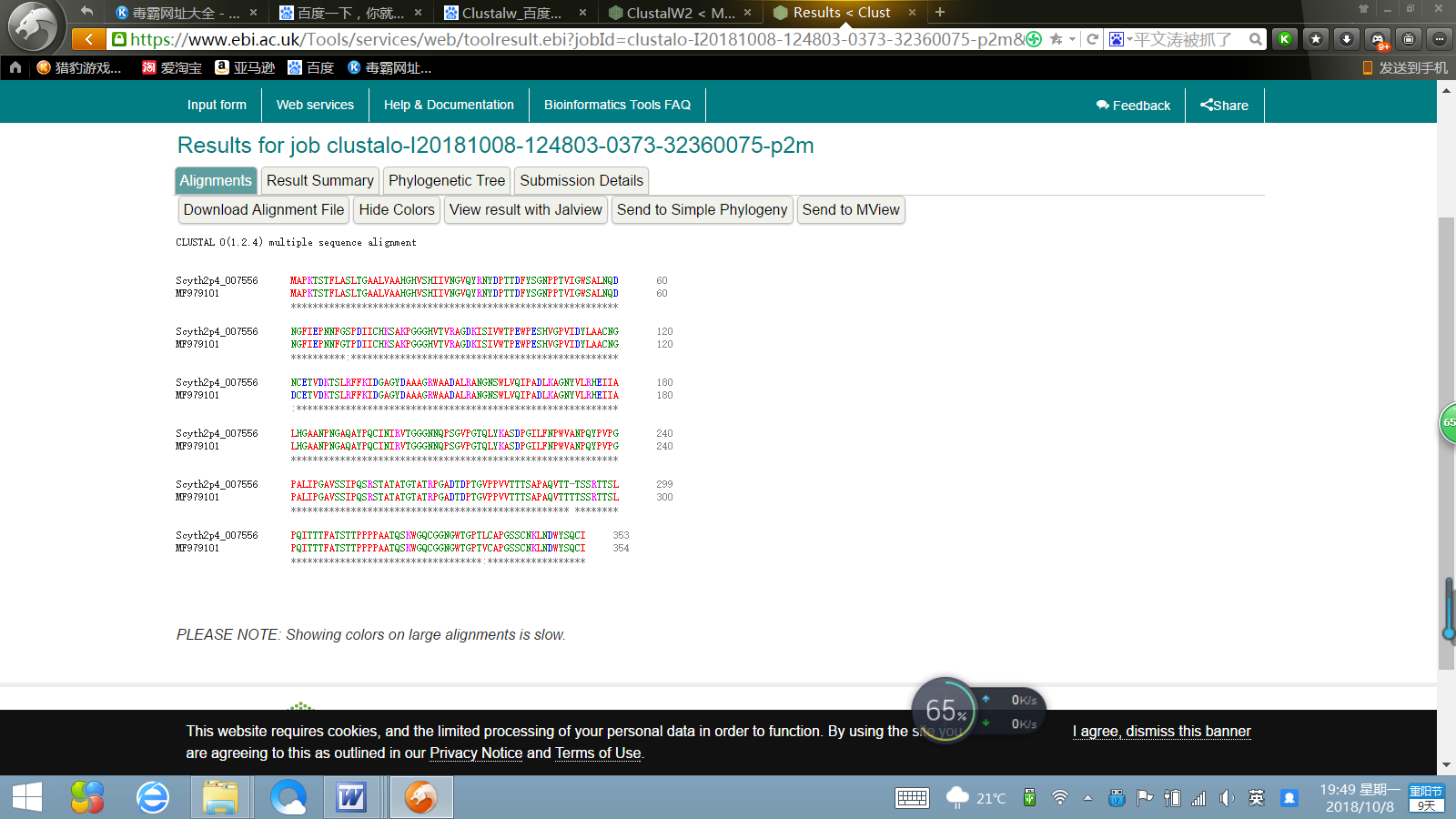


**▼**

**▼**

**▼**

**▼**

**Figure S2.** HiPMO1 N-terminal amino acid sequence analysis using LC-MS. LC-MS analysis of the digested CtPMO1 protein with trypsin reveals a peak *m/z* of 572.6396. The *m/z* value is 1/3 of the molecular weight of the peptide HGHVSHIIVNGVQYR, indicating that the *m/z* 572.6396 ion is triply charged (*z*=3). The extracted-ion chromatogram (XIC) of the peptide HGHVSHIIVNGVQYR was also shown.

**
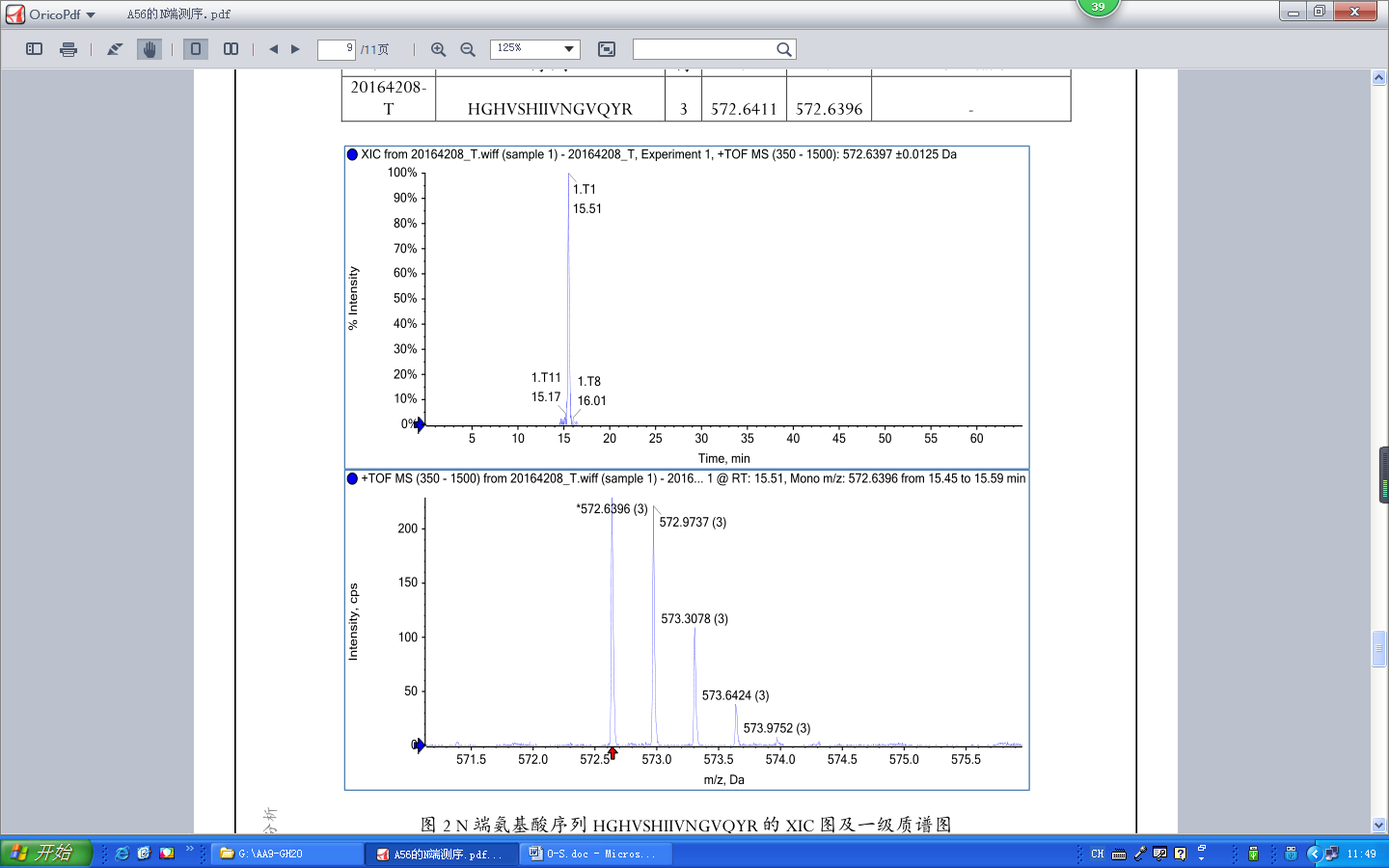
**

**TIC**

**MS**

**Figure S3.** HiPMO1 N-terminal amino acid sequence analysis using LC-MS/MS. LC-MS/MS analysis shows that fragmentation *m/z* values of the *m/z* 572.6408 ion agree with the molecular weight of the corresponding fragmentations of the peptide HGHVSHIIVNGVQYR. These data indicate that the N-terminal amino acid sequence of HiPMO1 is HGHVSHIIVNGVQYR.

**
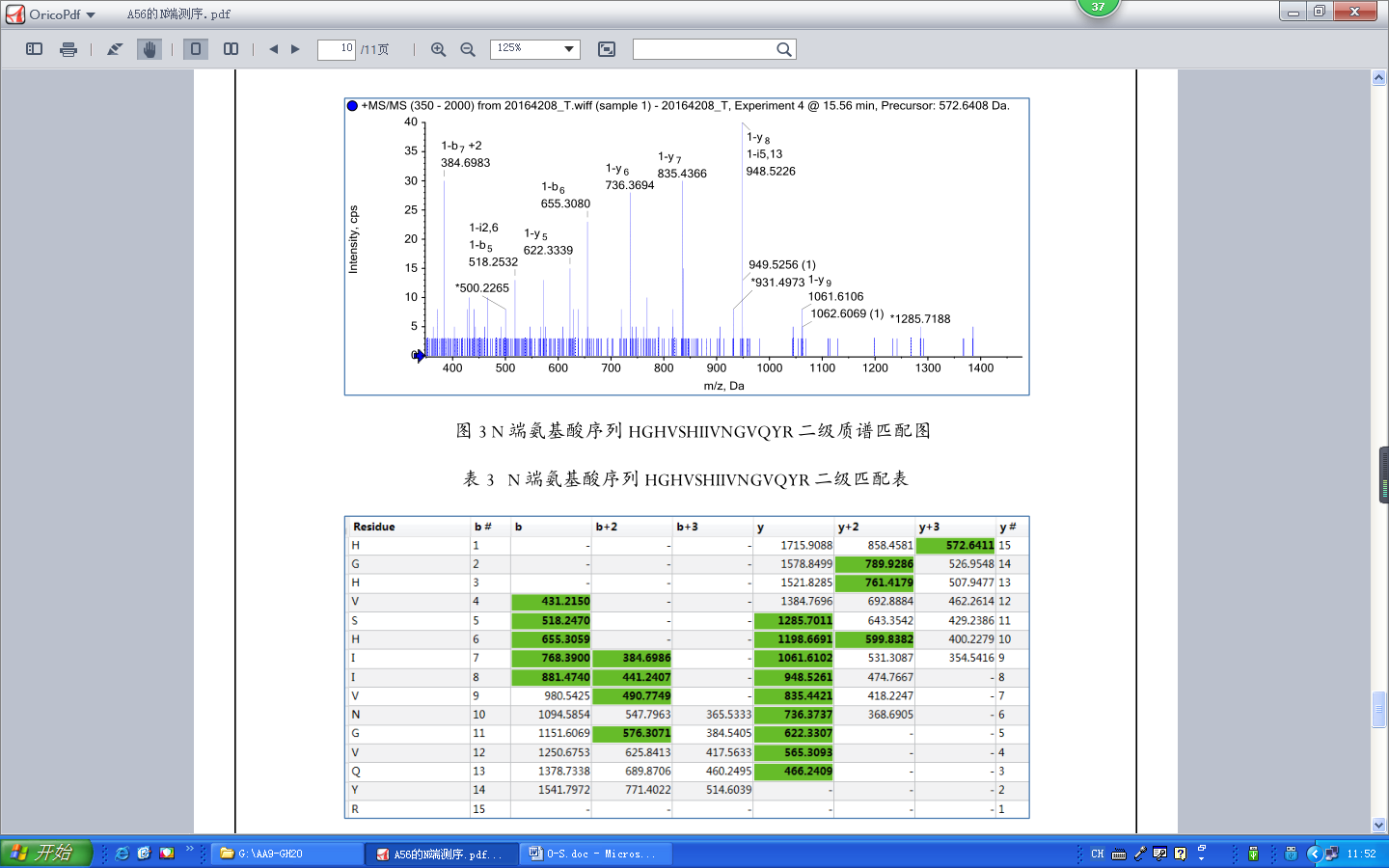
**

**MS/MS**

**
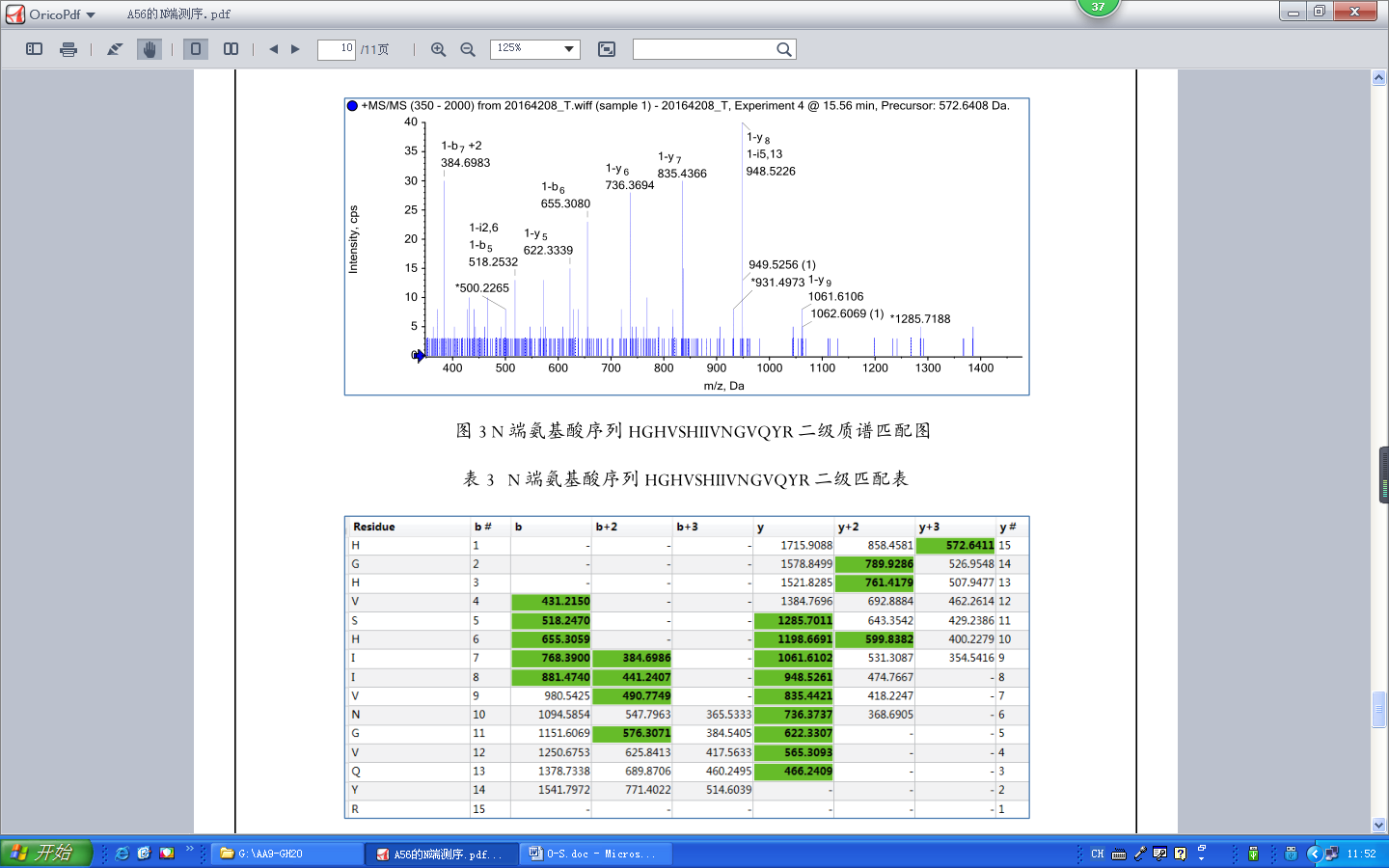
**

**Figure S4.** The analysis of MALDI-TOF-MS/MS of HiPMO1 reaction products. We selected the highest peak at *m/z* 525 from MALDI-TOF-MS analysis for MS/MS. MS/MS data were acquired on the mass *m/z* range of 100–550. We observed the various fragmentation ions of the main HiPMO1 C4 or C6 oxidized product (*m/z* 525).

**
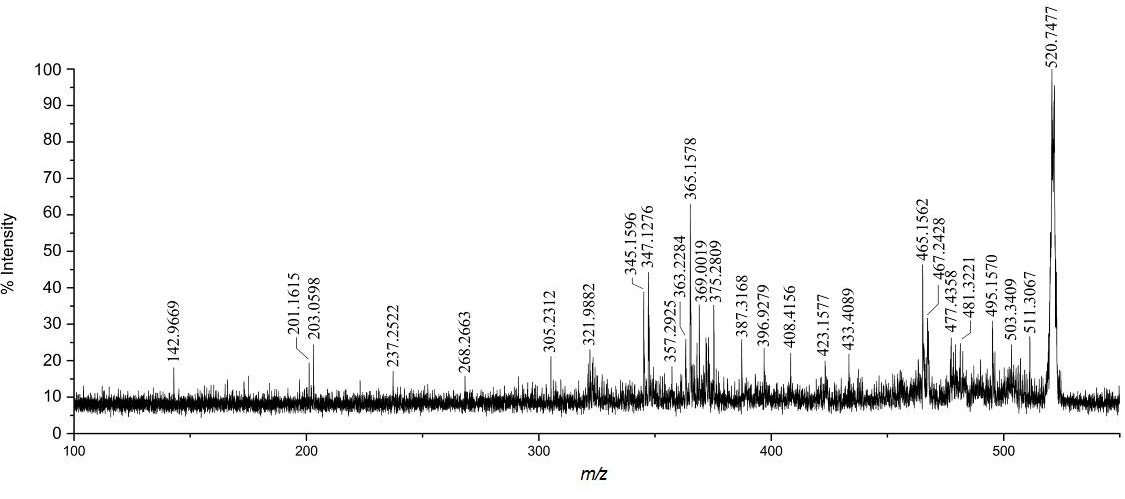
**

**Figure S5.** Molecular ion peaks of HiPMO1 reaction products hydrolyzed by beta-glucuronidase and beta-glucosidase by Full Scan LC-MS in positive mode. Most molecular ion peaks have an absolute intensity exceeding 1,000.


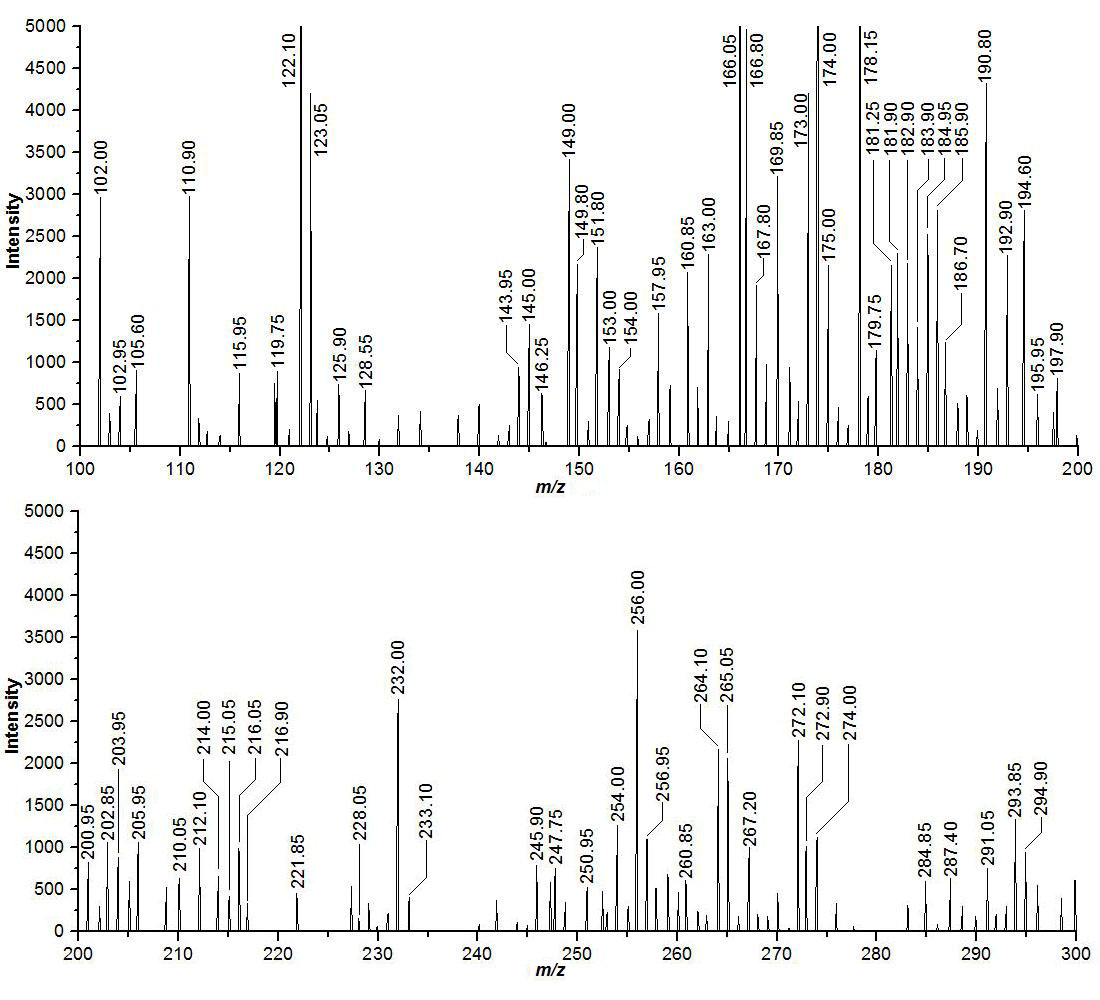


**Figure S6.** Molecular ion peaks of HiPMO1 reaction products hydrolyzed by beta-glucuronidase and beta-glucosidase by Full Scan LC-MS in negative mode. Most molecular ion peaks have an absolute intensity exceeding 200.


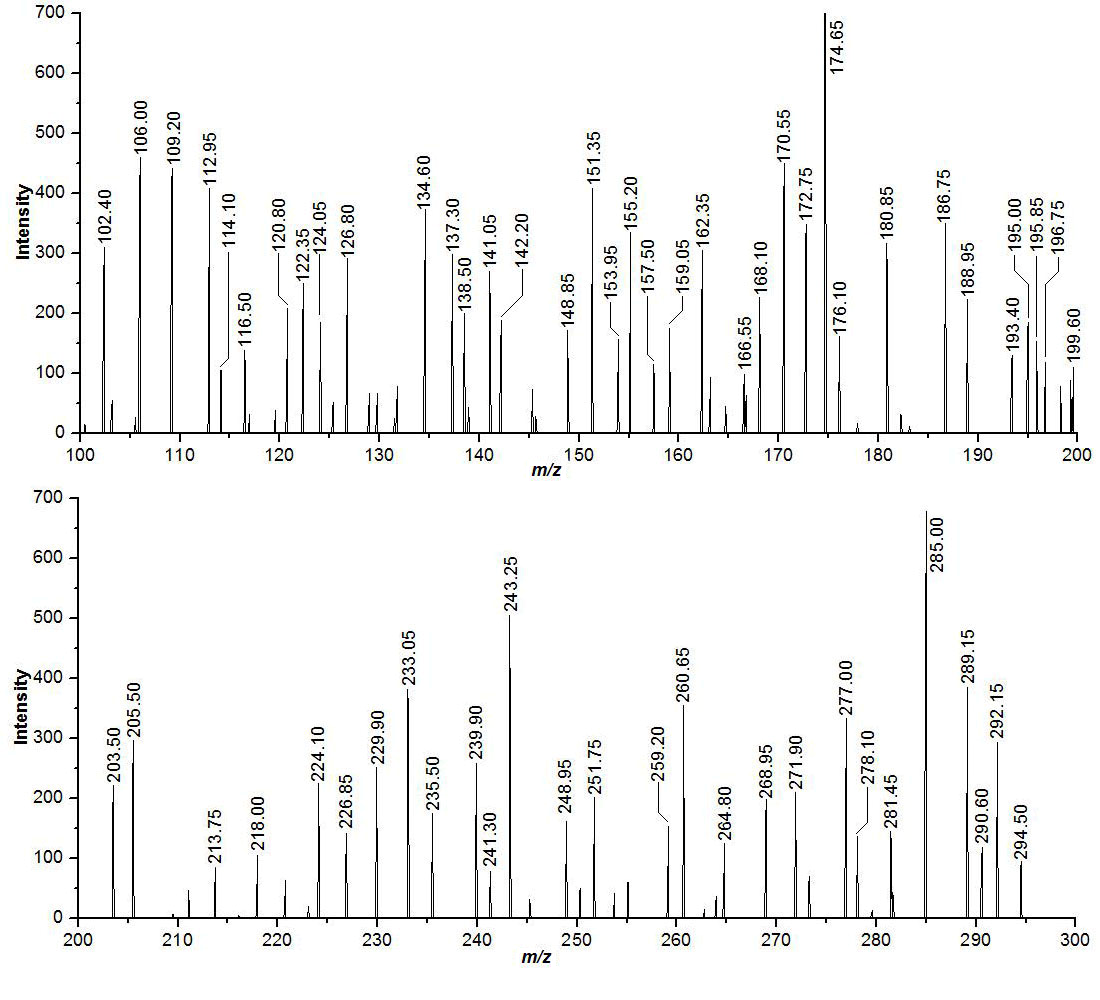


**Figure S7.** Analysis of CtPMO1 reaction products hydrolyzed by beta-glucuronidase and beta-glucosidase using SIM LC-MS/MS analysis. SIM LC-MS showing extracted ion chromatograms and the corresponding mass spectra of glucuronic acid and saccharic acid (saccharic acid lactone). In positive mode: saccharic acid (*m/z* 210+H^+^), saccharic acid lactone (*m/z* 192+H^+^) and glucuronic acid (*m/z* 194+H^+^).


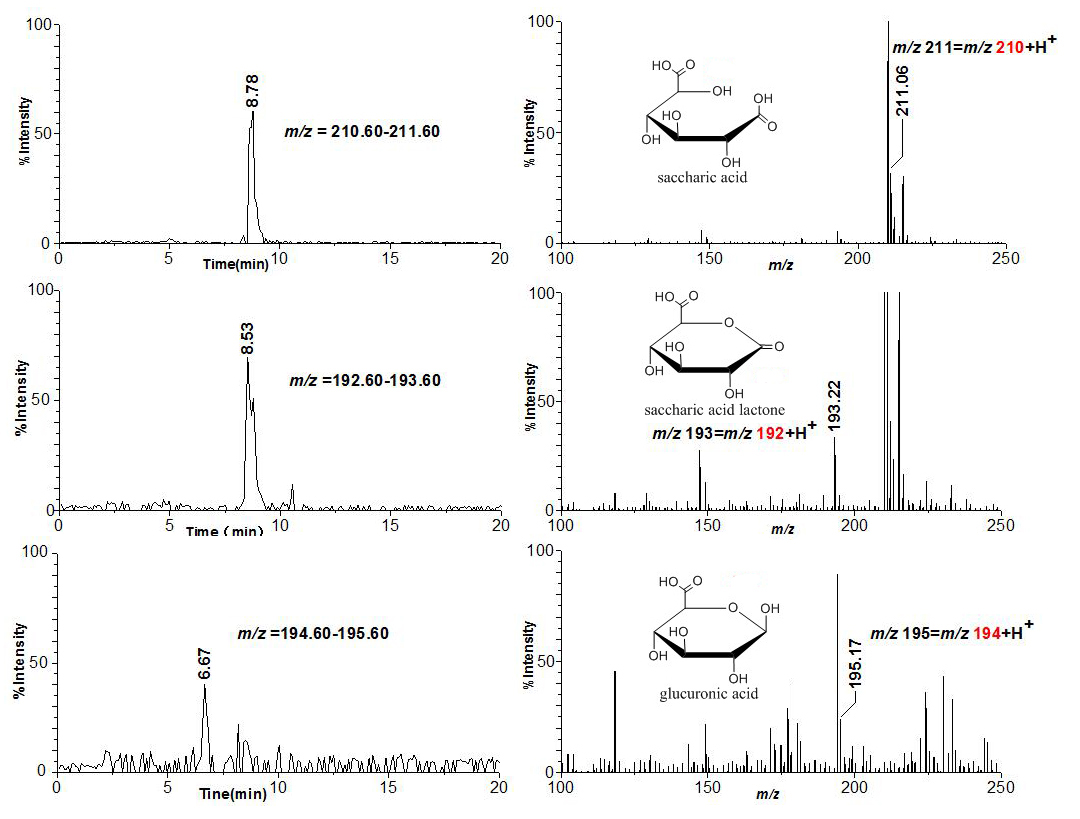


**Figure S8.** Analysis of CtPMO1 reaction products hydrolyzed by beta-glucuronidase and beta-glucosidase using HPAEC-PAD analysis. **(a)** **Qualitative** **analysis:** CtPMO1-CtPMO1 reaction products hydrolyzed by beta-glucuronidase and beta-glucosidase; CK-the control sample analyzed as above except without CtPMO1; Standard-glucose, gluconic acid, glucuronic acid, and saccharic acid. **(b) Quantitative analysis:** The rate of formation of gluconic acid, glucuronic acid and saccharic acid in CtPMO1 reaction products hydrolyzed with beta-glucuronidase and beta-glucosidase were quantified from standard curves of gluconic acid, glucuronic acid and saccharic acid using HPAEC-PAD analysis. The unit of rate was expressed in µg/µM·h, the amount (µg) of product formed per enzyme molecule (µM) per unit time (h). The control sample analyzed as above except without CtPMO1 (CK).

**(a)**

**
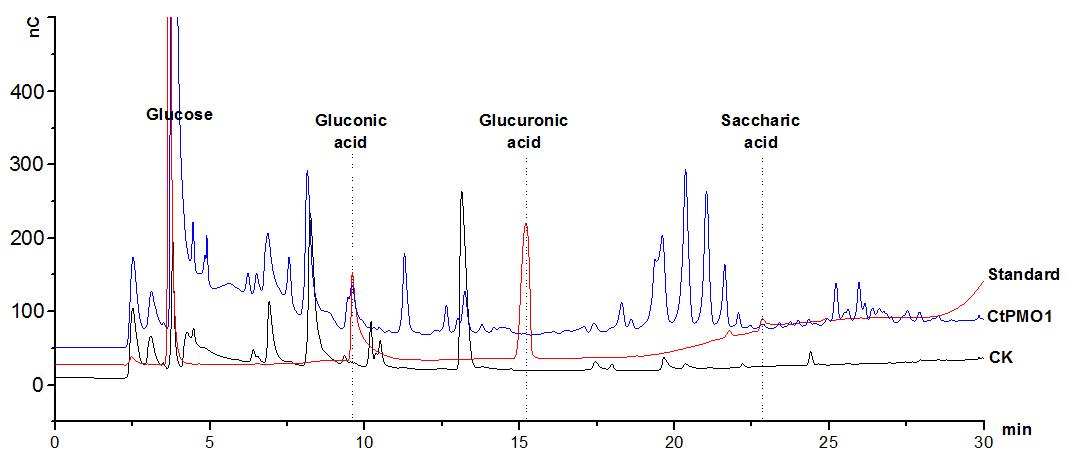
**

**(b)**

**
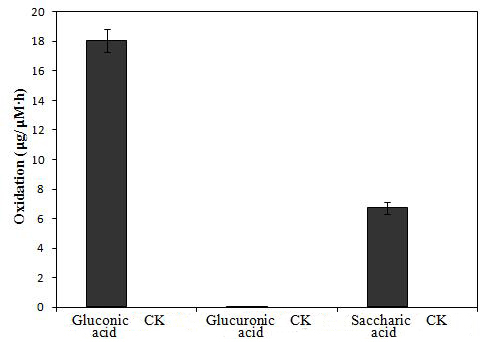
**

**Figure S9.** Sequence alignment of catalytic domains of HiPMO1 and *Thermoascus aurantiacus* TaGH61 (PDB ID: 2YET) using ClastalW2.


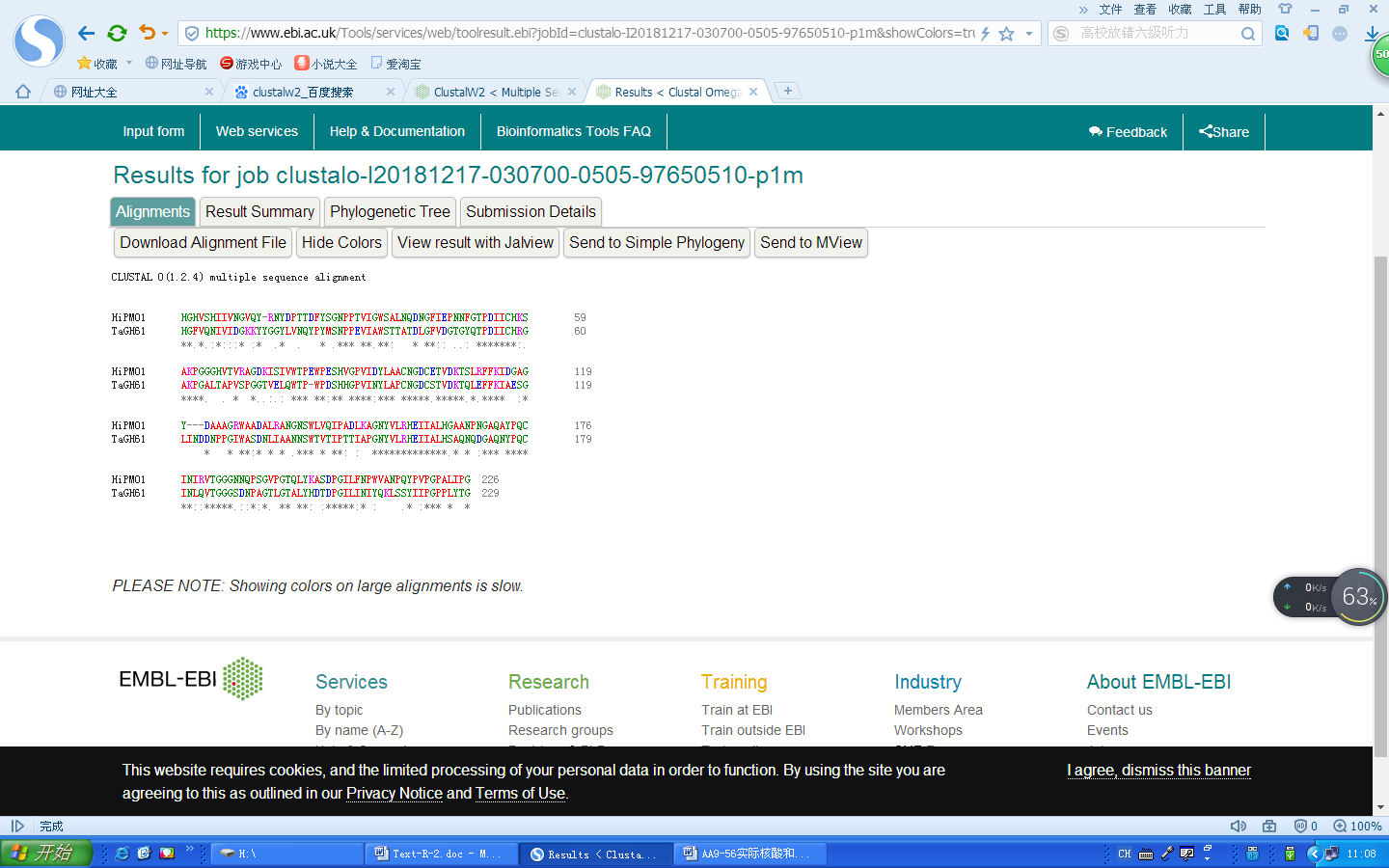


**Figure S10.** Homology model of the catalytic domain of HiPMO1 with  *Thermoascus aurantiacus* TaGH61 (PDB ID: 2YET) as a template using SWISS-MODEL. The globally conserved residues adjacent to the copper are colored in red. The aromatic and Ser86 residues are colored in blue. The copper ion is shown as an orange sphere.

**
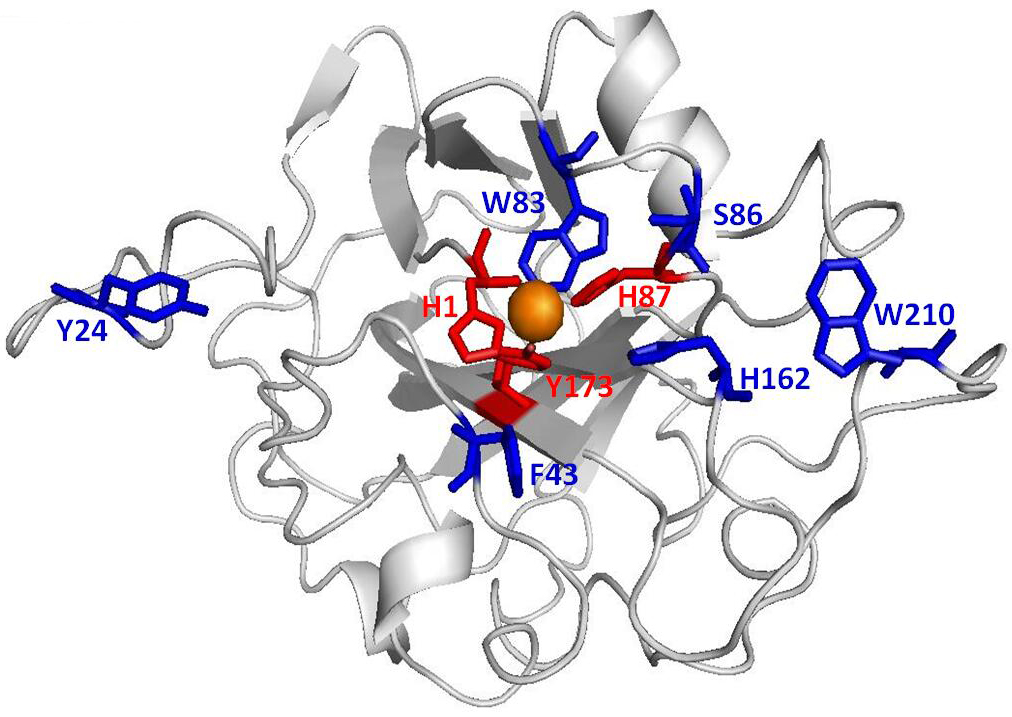
**

**Figure S11.** Docking study of HiPMO1 binding with cellopentaose using PyMOL. HiPMO1 model was aligned to LsAA9A:cellopentaose (PDB ID: 5NLS). The globally conserved residues adjacent to the copper are colored in red. The aromatic and Ser86 residues are colored in blue. The copper ion is shown as an orange sphere. The carbon atoms of cellopentaose are colored in green, and oxygen atoms of cellopentaose are colored in light red.

**
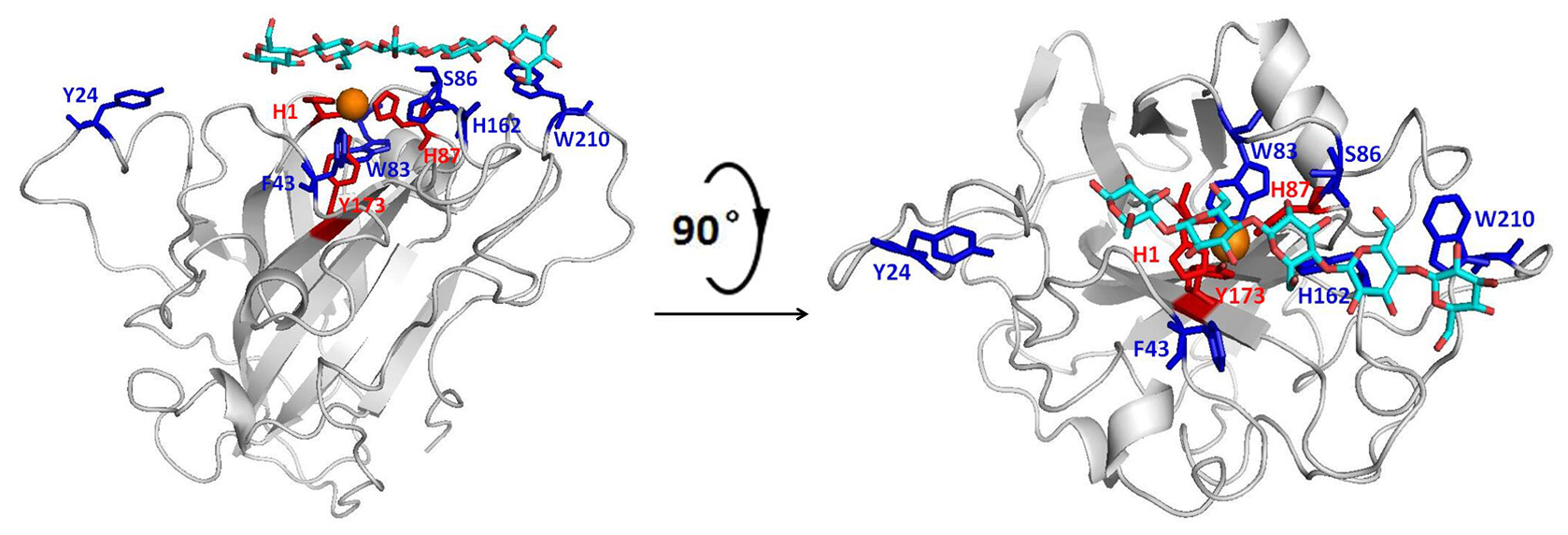
**

**Figure S12.** Speculative mechanism of C6 oxidation by HiPMO1. (**a**) Active sites of galactose oxidase (GO) and HiPMO1 [59, 63]. Four ligand atoms directly coordinating copper were shown. C6 oxygen atom of substrate (S) coordinating copper was also shown. (**b**) The mechanism of C6 oxidation by galactose oxidase (GO) [59, 63]. (**c**) Speculative mechanism of C6 oxidation by HiPMO1. In the presence of reductants (electron donors), HiPMO1-Cu(II)-H1 is first reduced to HiPMO1-Cu(I)-H1 (priming reduction), which reacts with O_2_ in the presence of substrate, resulting to generation of H_2_O_2_ and HiPMO1-Cu(II)-H1•. The resulting HiPMO1-Cu(II)-H1• reacts with substrate, leading to generation of RCHO or RCOOH. H1• denotes the His1 radical cofactor of HiPMO1, similar to the Tyr272 radical cofactor of galactose oxidase (GO)(Y272•).

**(a)**

**GO HiPMO1**

**
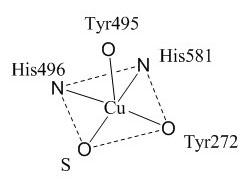

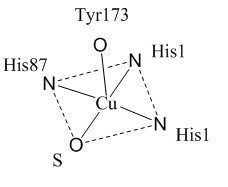
**

**(b)**

**
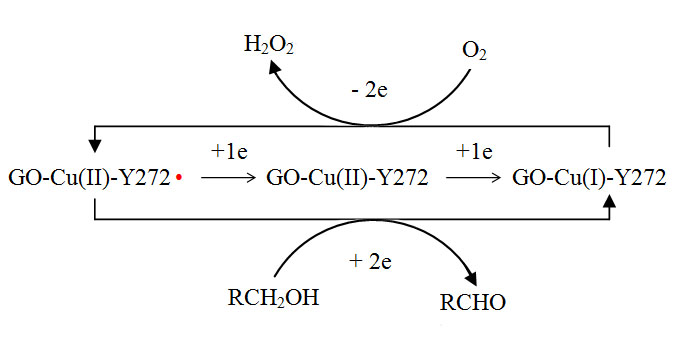
**

**(c)**

**
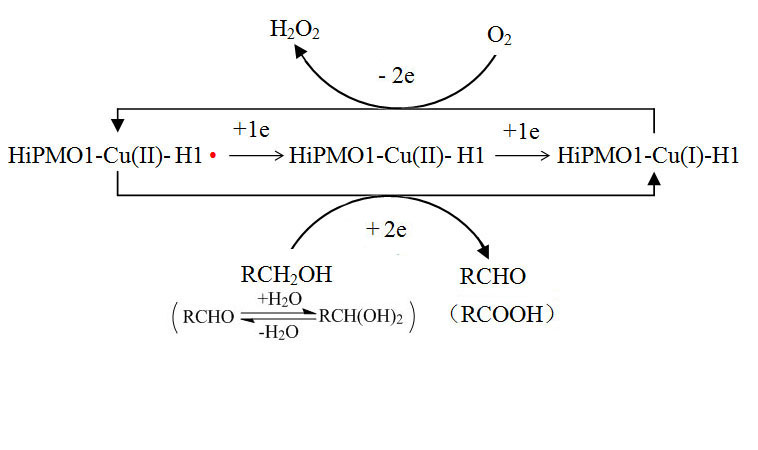
**

**Table S1.** List of primers used for PCR of the *Hipmo1* gene*.* The pair of oligonucleotide primers (D1 and D2) was synthesized based on the gene ([Scyth2p4_007556](http://genome.fungalgenomics.ca/new_gene_model_pages/gene_model_page.php?gmid=Scyth2p4_007556)) from the genomic sequencing of *H*. *insolens* ([www.fungalgenomics.ca](http://www.fungalgenomics.ca/)).

| Primers | Sequence | Purpose |
| --- | --- | --- |
| D1  D2  D3  D4 | 5’-ATGGCTCCCAAGACCTCGACGT-3’  5’-TTAGATGCACTGCGAGTACCAGT-3’  5’-AGGGGTATCTCTCGAGAAAAGACACGGCCATGTCAGCCA -3’  5’-GAGTTTTTGTTCTAGAGCGATGCACTGCGAGTACCAGT-3’ | ORF cDNA of HiPMO1  ORF cDNA of HiPMO1  Expression of HiPMO1  Expression of HiPMO1 |

[**Table S2**.](https://doi.org/10.1186/s13068-018-1269-7)The putative potential O-linked glycosylation sites of HiPMO1. A list of potential glycosylation sites showed their positions in the sequence and the prediction confidence scores. Only the sites with scores higher than 0.5 are predicted to be glycosylated.

| positions | scores | positions | scores |
| --- | --- | --- | --- |
| 5  20  21  25  30  35  51  59  68  76  80  86  104  108  109  138  182  190  195  201  229  230  234  236  237  239  241  243  245 | 0.0706695  0.120251  0.0913743  0.456291  0.242201  0.167365  0.130934  0.363719  0.273223  0.0539045  0.035829  0.0770685  0.023622  0.0375559  0.104577  0.176021  0.223012  0.721825  0.0801153  0.353233  0.868162  0.776211  0.964246  0.978911  0.957834  0.953781  0.943764  0.868281  0.959392 | 251  254  261  262  263  264  270  271  272  273  274  275  277  278  279  284  285  286  289  290  291  292  299  301  312  315  321  322  331 | 0.907698  0.960421  0.961639  0.98804  0.970199  0.973086  0.940838  0.959616  0.979707  0.938539  0.984512  0.981148  0.970413  0.975592  0.969193  0.950391  0.971324  0.955139  0.931994  0.987953  0.988056  0.980895  0.904911  0.524281  0.224509  0.486322  0.0805682  0.139255  0.00562688 |

**Table S3.** The analysis of fragmentation ions of the peak of DP_3_-2 (*m/z* 525) of HiPMO1 reaction products according to Additional file 1: Figure S4. Table S3 shows the type of fragmentation ions and the potential oxidized positions. Fragmentation ion types were nominated according to the methods previously described [29, 38].

| m/z | Potential fragmentation ion types | Potential products |
| --- | --- | --- |
| 521  511  503  481  477  467  465  433  423  397  387  375  369  365  363  347  345  305  237  203  201  143 | Y_3_+H_2_O+H^+^-2  C_3_+H_2_O+H^+^-2  ^3,4^X_3_+Na^+^+H_2_O-2-2  Y_3_+H^+^-2  C_3_+H^+^-2  ^1,5^A_3_+Na^+^  H_3_-2+Na^+^  ^2,4^X_3_/^0,2^A_3_+Na^+^  ^2,4^X_3_+Na^+^-2  ^0,2^A_3_+Na^+^-2  ^0,3^A_3_+Na^+^-2-2  ^0,3^X_3_+Na^+^-2-2  ^2,4^A_3_+Na^+^+H_2_O-2  ^0,2^X_3_+Na^+^+H_2_O-2  ^3,5^A_3_-2+H^+^  ^0,2^X_3_+Na^+^-H_2_O-2  ^2,4^A_3_+Na^+^-H_2_O-2  ^0,1^X_3_+Na^+^-2  ^3,4^A_3_+Na^+^-2  Z_2_/B_2_+2Na^+^-H^+^  ^1,5^X_3_+H^+^-2  C_2_/Y_2_+Na^+^  Y_2_+Na^+^-2  C_2_+Na^+^-2  Z_2_/B_2_+Na^+^  Z_2_+Na^+^-2  B_2_+Na^+^-2  ^0,2^A_2_+Na^+^  ^2,4^X_2_+Na^+^  ^3,5^A_2_+H^+^  C_1_/Y_1_+Na^+^  C_1_+Na^+^-2  Y_1_+Na^+^-2  ^2,4^X_1_/^0,2^A_1_+Na^+^ | C6 oxidized products  C6 or C4 oxidized products  C6 oxidized products  C6 oxidized products  C6 or C4 oxidized products  non-oxidized products  C6 oxidized products  non-oxidized products  C6 oxidized products  C6 or C4 oxidized products  C6/C4 or C6/C6-oxidized products  C6/C6-oxidized products  C6 or C4 oxidized products  C6 oxidized products  C6 or C4 oxidized products  C6 oxidized products  C6 or C4 oxidized products  C6 oxidized products  C6 or C4 oxidized products  non-oxidized products  C6 oxidized products  non-oxidized products  C6 oxidized products  C6 or C4 oxidized products  non-oxidized products  C6 oxidized products  C6 or C4 oxidized products  non-oxidized products  non-oxidized products  non-oxidized products  non-oxidized products  C6 or C4 oxidized products  C6 oxidized products  non-oxidized products |
